# Sensitivity of Air Pollution Exposure and Disease Burden to Emission Changes in China Using Machine Learning Emulation

**DOI:** 10.1029/2021GH000570

**Published:** 2022-06-01

**Authors:** Luke Conibear, Carly L. Reddington, Ben J. Silver, Ying Chen, Christoph Knote, Stephen R. Arnold, Dominick V. Spracklen

**Affiliations:** ^1^ School of Earth and Environment Institute for Climate and Atmospheric Science University of Leeds Leeds UK; ^2^ College of Engineering Mathematics and Physical Sciences University of Exeter Exeter UK; ^3^ Faculty of Medicine University of Augsburg Augsburg Germany

**Keywords:** emulator, machine learning, air quality, health impact assessment, China, particulate matter

## Abstract

Machine learning models can emulate chemical transport models, reducing computational costs and enabling more experimentation. We developed emulators to predict annual−mean fine particulate matter (PM_2.5_) and ozone (O_3_) concentrations and their associated chronic health impacts from changes in five major emission sectors (residential, industrial, land transport, agriculture, and power generation) in China. The emulators predicted 99.9% of the variance in PM_2.5_ and O_3_ concentrations. We used these emulators to estimate how emission reductions can attain air quality targets. In 2015, we estimate that PM_2.5_ exposure was 47.4 μg m^−3^ and O_3_ exposure was 43.8 ppb, associated with 2,189,700 (95% uncertainty interval, 95UI: 1,948,000–2,427,300) premature deaths per year, primarily from PM_2.5_ exposure (98%). PM_2.5_ exposure and the associated disease burden were most sensitive to industry and residential emissions. We explore the sensitivity of exposure and health to different combinations of emission reductions. The National Air Quality Target (35 μg m^−3^) for PM_2.5_ concentrations can be attained nationally with emission reductions of 72% in industrial, 57% in residential, 36% in land transport, 35% in agricultural, and 33% in power generation emissions. We show that complete removal of emissions from these five sectors does not enable the attainment of the WHO Annual Guideline (5 μg m^−3^) due to remaining air pollution from other sources. Our work provides the first assessment of how air pollution exposure and disease burden in China varies as emissions change across these five sectors and highlights the value of emulators in air quality research.

## Key Points

- We developed emulators to predict air quality and chronic health impacts across China from changes in emissions (based on 2015 data)
- Annual−mean PM_2.5_ exposure and the associated disease burden were most sensitive to changes in industrial and residential emissions
- Removing emissions from five key sectors in China does not attain the World Health Organization Annual Guideline due to remaining pollution from other sources

## 1 Introduction

Air pollution exposure is a key public health problem in China (GBD, 2019 Risk Factors Collaborators, 2020; Yin et al., 2020). In recent years, particulate air quality has improved, primarily attributed to reductions in anthropogenic emissions (Cheng et al., 2019; Ding et al., 2019; Li et al., 2020, 2019; Silver, Conibear, et al., 2020; Silver, He et al., 2020; Silver et al., 2018; Zhai et al., 2019). However, the loss of healthy life from air pollution exposure remains substantial (Conibear, Reddington, Silver, Knote, et al., 2021; Silver, Conibear, et al., 2020; Zhao et al., 2018) and further emission reductions are required to improve air quality.

Numerical chemical transport models (CTMs) are useful for simulating air quality and its driving processes. CTMs discretize the atmosphere into cells on a three‐dimensional grid and compute complex processes, mechanisms, laws, and parameterisations at high temporal resolutions on this grid (Brasseur & Jacob, 2017). However, these complex CTMs have high computational costs. To reduce costs, some approaches are to reduce the model complexity, reduce the model resolution or precision (Palmer, 2015), reduce the number of experiments, or to use simplified models. To reduce computational demand, a wide range of reduced−complexity or reduced−form air quality models have been developed to simplify these complex processes (Buonocore et al., 2014; Carnevale et al., 2009; Foley et al., 2014; Heo, Adams & Gao, 2016a, 2016b; Henze et al., 2007; Seinfeld & Pandis, 2016; Tessum et al., 2017). For example, InMAP (Intervention Model for Air Pollution) is a reduced−form air quality model that decreases computational costs via simplified representations of atmospheric processes (Tessum et al., 2017). In contrast, the emulators are statistical machine learning models that decrease computational costs via mapping specific associations. Emulators learn these specific input−output associations from full CTM simulations. These emulators are often designed using Gaussian process regressors (O’Hagan, 2006; Rasmussen & Williams, 2006), due to their flexibility, accuracy, and skill with smaller data sets. Emulators are computationally expensive to build as their training data requires many CTM runs, though they are substantially cheaper to run once built enabling many more experiments to be explored.

Previous studies have used emulators for prediction of air quality (Beddows et al., 2017; Chen et al., 2020; Conibear, Reddington, Silver, Chen, et al., 2021), weather (Chantry, Christensen, et al., 2021; Gettelman et al., 2021; Weyn et al., 2019), and climate (Beusch et al., 2020; Chantry, Christensen, et al., 2021; Holden et al., 2019; Ott et al., 2020; Scher, 2018; Tran et al., 2016). For example, the air quality prediction studies used emulators to analyze the drivers of an ozone (O_3_) pollution episode in the United Kingdom (Beddows et al., 2017) and emission reduction strategies in India (Chen et al., 2020). Some studies have used machine learning models to represent processes, such as radiation (Chevallier et al., 2000; Krasnopolsky et al., 2005), convection (Beucler et al., 2020; Brenowitz & Bretherton, 2018; Gentine et al., 2018; Han et al., 2020; O’Gorman & Dwyer, 2018; Rasp et al., 2018; Yuval & O’Gorman, 2020), chemistry (Ivatt & Evans, 2020; Keller & Evans, 2019; Kelp et al., 2020; Kelp et al., 2022), physics (Chantry, Hatfield et al., 2021; Harder et al., 2021; Hatfield et al., 2021; Hughes et al., 2018; Krasnopolsky, 2020; Silva et al., 2021), and land surface models (Dagon et al., 2020). Many studies have used emulators to explore uncertainties and sensitivities (Aleksankina et al., 2019; Carslaw et al., 2013; Chang et al., 2016; Johnson et al., 2015; Lee et al., 2012; Lee et al., 2011; Lee et al., 2016; McCoy et al., 2020; Nicely et al., 2020; Ryan & Wild, 2021; Ryan et al., 2018; Rybarczyk & Zalakeviciute, 2018; Salter et al., 2018; Watson‐Parris, 2021; Watson‐Parris et al., 2020; Watson‐Parris et al., 2021; Wild et al., 2020).

In our previous work, we developed emulators to predict winter (January 2015) ambient fine particulate matter (PM_2.5_) concentrations from emission changes across China (Conibear, Reddington, Silver, Chen, et al., 2021). Here, we further develop these emulators for annual exposure (2015) to multiple air pollutants (PM_2.5_ and O_3_) and to assess the chronic health impacts. To our knowledge, this is the first study using emulators to predict long−term (annual) air quality and the public health benefits attributed to different emission control strategies in China.

## 2 Methods

### 2.1 Simulator

Simulations were conducted using WRFChem (Weather Research and Forecasting model online–coupled with Chemistry) version 3.7.1 (Grell et al., 2005; Skamarock et al., 2008). Each simulation was for the whole of 2015 with one–month spin–up over China at 30 km (0.3°) horizontal resolution. There were 50 simulations for the training data and five additional simulations for the test data. The simulations differed only in the scaling of the anthropogenic emissions over China, determined from separate maxi−min Latin hypercube space–filling designs (Tables S1 and S2 in Supporting Information S1). The version of WRFChem used here was described and evaluated in our previous work (Conibear, Reddington, Silver, Chen, et al., 2021; Conibear, Reddington, Silver, Knote, et al., 2021; Reddington et al., 2019; Silver, Conibear, et al., 2020).

Sectoral and speciated anthropogenic emissions inside China were from the MEIC (Multi–resolution Emission Inventory for China) emission inventory for 2015 at 0.25° × 0.25° horizontal resolution (Li, Liu, et al., 2017; Li, Zhang, et al., 2017; MEIC Research Group & Tsinghua University, 2019; Zheng et al., 2018). Sectoral and speciated anthropogenic emissions outside China were from EDGAR−HTAP (Emission Database for Global Atmospheric Research with Task Force on Hemispheric Transport of Air Pollution) version 2.2 for 2010 at 0.1° × 0.1° horizontal resolution (Janssens‐Maenhout et al., 2015). Anthropogenic emissions are largest over East, North, and South China, as well as across South Asia (Figures S1−S4 in Supporting Information S1). A diurnal cycle was applied to the anthropogenic emissions (Qi et al., 2017; Zheng et al., 2017).

Gas phase chemistry was simulated using the extended MOZART (Model for Ozone and Related Chemical Tracers) scheme (Emmons et al., 2010; Hodzic & Jimenez, 2011; Knote et al., 2014). Aerosol physics and chemistry was simulated using the updated MOSAIC (Model for Simulating Aerosol Interactions and Chemistry) scheme with aqueous chemistry (Alma Hodzic & Knote, 2014; Zaveri et al., 2008). Secondary organic aerosol (SOA) formation was based on an updated volatility basis set mechanism (Knote et al., 2015).

We evaluated the simulator (WRFChem) against PM_2.5_ and O_3_ measurements from 1,633 sites (Jin et al., 2020; Silver et al., 2018). The normalized mean bias factor (NMBF) and the normalized mean absolute error factor (NMAEF) were used to evaluate the simulator (Yu et al., 2006). For example, a NMBF of −0.5 means that the simulator underestimated observations by 50% on average, and a NMAEF of 0.5 means that the simulator had an absolute gross error of 0.5 times the mean observation. Here, the simulator underestimated annual−mean PM_2.5_ concentrations (NMBF = −0.05 and NMAEF = 0.18) and overestimated maximum 6−monthly−mean daily−maximum 8−hour (6mDM8h) O_3_ concentrations (NMBF = 0.39 and NMAEF = 0.40) across China (Figure S5 in Supporting Information S1). To provide the closest match with observations, we tuned the PM_2.5_ and O_3_ concentrations. Tuning was completed by scaling the model to match observations by prefecture if measurements were available, otherwise scalings were applied by province (administrative division). The tuned model had reduced bias and error for both annual−mean PM_2.5_ concentrations (NMBF = 0.02 and NMAEF = 0.10) and 6mDM8h O_3_ concentrations (NMBF = 0.03 and NMAEF = 0.11) across China. The scaling allowed us to accurately predict the spatial pattern and magnitude of PM_2.5_ and O_3_ concentrations across China.

### 2.2 Emulator

We developed emulators to make computationally efficient predictions of the WRFChem model, as described in our previous work (Conibear, Reddington, Silver, Chen, et al., 2021). Here, the emulator approach from our previous work was extended from January 2015 to the year of 2015, for O_3_ concentrations, and the air pollution disease burden. The emulator workflow is summarized in Figure S6 in Supporting Information S1.

The emulators predict air quality across China from fractional changes in anthropogenic emissions. The emulator inputs were anthropogenic emissions from the residential (RES), industrial (IND), land transport (TRA), agricultural (AGR), and power generation (ENE) sectors. For the emulator inputs, all species from each sector were scaled between 0%–150%. These emulator inputs were from simulator data of 50 training runs and 5 test runs, based on separate maxi−min Latin hypercube space–filling designs. The training data was designed to cover all of the input distributions. The emulators were trained on the raw simulation data, before the control scaling factors were applied for tuning.

Individual emulators were developed for each output of annual−mean PM_2.5_ concentrations and 6mDM8h O_3_ concentrations, and each WRFChem grid cell across China (15,278 grid cells in total) to capture the spatial distribution of each pollutant. These outputs were chosen as they are the metrics used in the health impact assessment. This meant we developed a total of 30,556 separate emulators. The emulators are based on annual average values and do not have information of finer time scales.

The optimized emulator designs included an input preprocessor (Yeo & Johnson, 2000) and a Gaussian process regressor with a Matern 5/2 kernel (Conibear, 2021). Gaussian process regressors update a prior function over the inputs to a posterior function including observations (i.e., the training data) (Rasmussen & Williams, 2006). Bayesian inference is then used to sample from this posterior function to produce the Gaussian output. Gaussian process regressors notice trends well when similar inputs have similar outputs, and are flexible and accurate with smaller data sets. Our emulators were based on Gaussian process regressors as they are accurate with only a few training samples. This was a key limitation as each training sample is an annual CTM simulation. Other emulator design options, such as deep neural networks, often require much larger training datasets to avoid overfitting the limited data (Watson−Parris, 2021). Recent developments in machine learning architectures may overcome this limitation to improve emulator accuracy and scope, for example, with deep neural architecture search (Kasim et al., 2022).

The creation of the simulation data (i.e., 55 annual WRFChem runs) was computationally expensive, using 320 CPUs (Central Processing Units, Intel Xeon Gold 6,138) for approximately 1 year of wall time. The training of the emulators used 150 CPUs for approximately 1 hr. Using the emulators per prediction run on the order of seconds on 1 CPU. Hence, the key bottleneck is simulating the atmosphere using complex numerical models. Reducing the computational burden of this step is an important area of future research.

The emulators were specific to the data they were trained on. The emulators make predictions based on associational knowledge, rather than explanatory knowledge (Deutsch, 2012; Pearl, 2019). The emulators were used to predict air quality concentrations for all emission configurations within a 0%–150% matrix of emission scaling factors at 20% increments (32,768 emission configurations).

**Figure 1:**
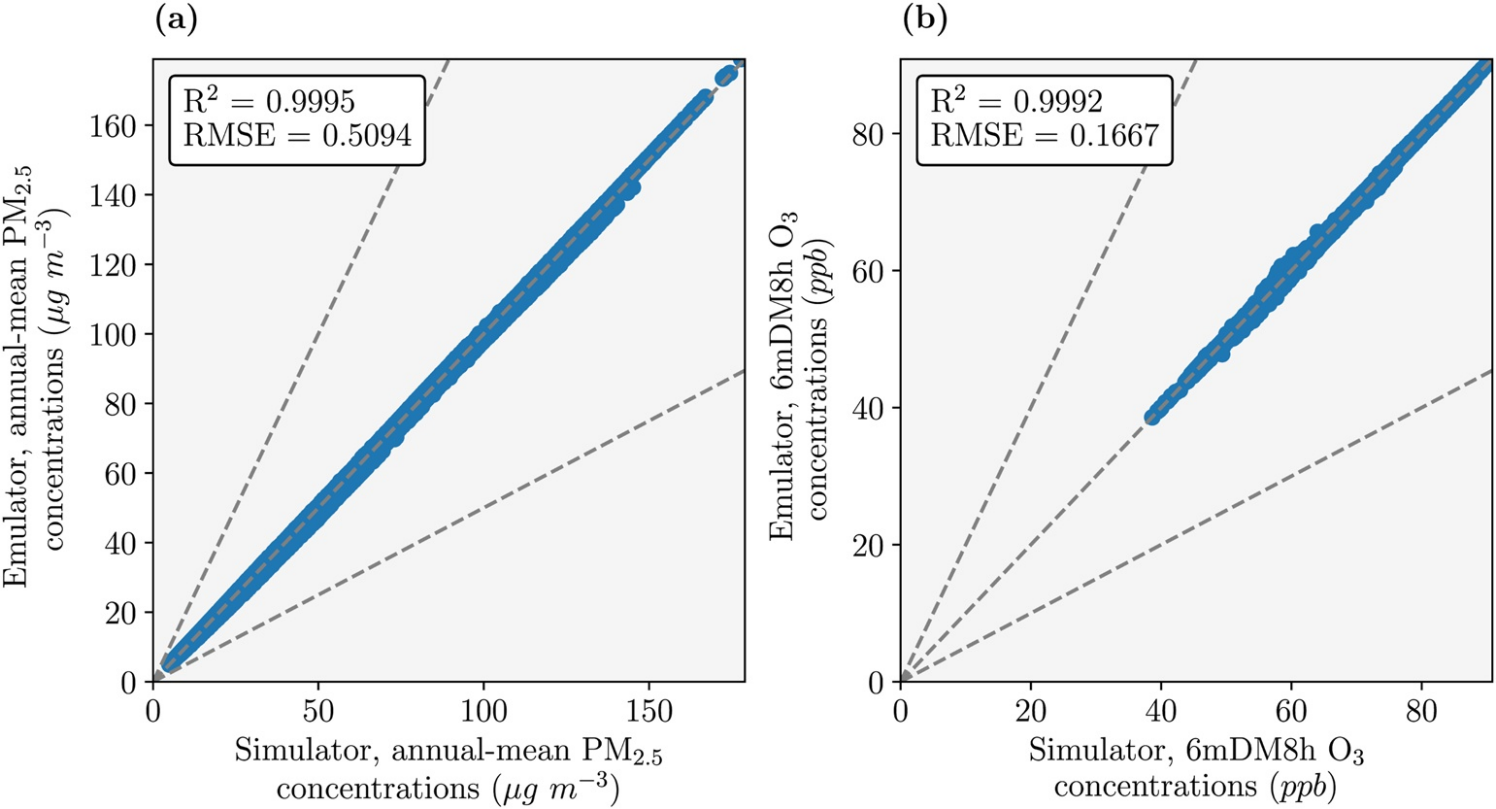
Evaluation of emulators on the unseen test data for concentrations of (a) fine particulate matter (PM_2.5_, annual−mean) and (b) ozone (O_3_, maximum 6−monthly−mean daily−maximum 8−hour, 6mDM8h). Evaluation metrics used were the coefficient of determination (R^2^) and the root mean squared error (RMSE).

Figure 1 shows the evaluation of the emulators on the unseen test data. For PM_2.5_ concentrations, the emulators have a coefficient of determination (R^2^) value of 0.9995 and a root mean squared error (RMSE) value of 0.5094 μg m^−3^. For O_3_ concentrations, the emulators have an R^2^ value of 0.9992 and a RMSE value of 0.1667 ppb. This means that the emulators can accurately predict 99.9% of the variance in both PM_2.5_ and O_3_ concentrations for any similar emission configuration.

### 2.3 Health Impact Assessment

The health impact assessment estimated the disease burden attributable to PM_2.5_ and O_3_ exposure using population attributable fractions (PAF) of relative risk (RR). Exposure variations were used to predict associated outcome variations.

The exposure to annual−mean PM_2.5_ (z) per grid cell was relative to the counterfactual exposure level of 2.4 μg m^−3^ (cf.) where no excess risk was assumed (Equation 1). The RR for a specific exposure and population age group was estimated through the GEMM (Global Exposure Mortality Model) (Burnett et al., 2018). The RR was a function of the parameters θ, α, μ, and ν (Equation 2) as defined in Supplementary Table 2 of Conibear, Reddington, Silver, Knote, et al. (2021). We used the GEMM for non−accidental mortality (non−communicable disease, NCD, plus lower respiratory infections, LRI), using parameters that included the China cohort, with age−specific modifiers for adults over 25 years of age in five−year intervals. The GEMM functions have mean, lower, and upper uncertainty intervals. The PAF was estimated as a function of the RR and the population count (P, Equation 3).

(1) $$z=\max\left(0,PM_{2.5}-cf\right)$$

(2) $$\text{RR}\left(z,\text{age}\right)=e^{\left\{\theta \frac{\log\left(1+\frac{z}{\alpha }\right)}{1+e^{\left(\frac{\mu -z}{\nu }\right)}}\right\}}$$

(3) $$\text{PAF}=P\times \left(1-\frac{1}{\text{RR}\left(z,\text{age}\right)}\right)$$

The health impact assessment for O_3_ exposure followed the methodology of the Global Burden of Diseases, Injuries, and Risk Factors Study (GBD) for 2017 (GBD, 2017 Risk Factor Collaborators, 2018). The exposure to O_3_ (z) per grid cell was calculated as the change in 6mDM8h O_3_ concentrations relative to the counterfactual exposure level of 35.7 ppb (cf.) where no excess risk was assumed (Equation 4) (Turner et al., 2016). The 6mDM8h metric was calculated by quantifying 24 separate 8−hour rolling mean O_3_ concentrations, finding the maximum of these each day, creating 12 separate 6−monthly means to account for seasonal variations, and finding the maximum of these over the year. The PAF was a function of the hazard ratio (HR), which was 1.06 (95UI: 1.02–1.10) for chronic obstructive pulmonary disease (COPD), based on data from five epidemiological cohorts (Equation 5) (GBD, 2017 Risk Factor Collaborators, 2018).

(4) $$z=\max\left(0,O_{3}-cf\right)$$

(5) $$\text{PAF}=P\times \left(1-e^{z\frac{\log HR}{10}}\right)$$

Premature mortality (MORT), years of life lost (YLL), and years lived with disability (YLD) per exposure, health outcome, age bracket, and grid cell were estimated as a function of the PAF and the corresponding baseline mortality and morbidity rate (I_MORT_, I_YLL_, and I_YLD_) following Equations (6), (7), (8), respectively. Disability−adjusted life years (DALYs) were estimated as the total of YLL and YLD (Equation 9). The rates of MORT, YLL, YLD, and DALYs were calculated per 100,000 people.

(6) $$MORT=PAF\times I_{MORT}$$

(7) $$YLL=PAF\times I_{YLL}$$

(8) $$YLD=PAF\times I_{YLD}$$

(9) DALYs=YLL+YLD

The United Nations adjusted population count data set for 2015 at 0.25° × 0.25° resolution was obtained from the Gridded Population of the World, Version 4 (Center for International Earth Science Information Network & NASA Socioeconomic Data and Applications Center, 2016). Population age composition was taken from the GBD2017 for 2015 for adults of 25–80 years of age in 5−year intervals, and for 80 years plus (Global Burden of Disease Study, 2017, 2018). Cause−specific (NCD, LRI, and COPD) baseline mortality and morbidity rates were taken from the GBD2017 for 2015 for MORT, YLL, and YLD for each age bracket (Institute for Health Metrics and Evaluation, 2020).

Shapefiles were used to aggregate results at the country, province, and prefecture level (Hijmans et al., 2020). Regional groupings were applied as follows: North China (Beijing, Tianjin, Hebei, Shanxi, and Inner Mongolia), North East China (Liaoning, Jilin, and Heilongjiang), East China (Shanghai, Jiangsu, Zhejiang, Anhui, Fujian, Jiangxi, and Shandong), South Central China (Henan, Hubei, Hunan, Guangdong, Guangxi, Hainan, Hong Kong, and Macau) including the Guangdong−Hong Kong−Macau Greater Bay Area (GBA), South West China (Chongqing, Sichuan, Guizhou, Yunnan, and Tibet), and North West China (Shaanxi, Gansu, Qinghai, Ningxia, and Xinjiang), and the GBA individually.

Uncertainty intervals at the 95% confidence level were estimated using the derived uncertainty intervals from the exposure−outcome associations, baseline mortality and morbidity rates, and population age fractions. Health impact assessments of the disease burden associated with air pollution exposure have many uncertainties (Nethery & Dominici, 2019).

## 3 Results and Discussion

In the results and discussion, PM_2.5_ concentrations are ambient annual−means and O_3_ concentrations are ambient 6mDM8h. Exposures are population−weighted concentrations. Air quality standards for O_3_ concentrations in units of μg m^−3^ are converted to ppb using the conversion factor of one ppb being approximately equal to 2 μg m^−3^ (Fleming et al., 2018).

### 3.1 Emulated Baseline PM_2.5_ and O_3_ Exposure and Disease Burden for 2015

Baseline (i.e., for 2015 with all emission sectors at 100%) PM_2.5_ exposure in China is 47.4 μg m^−3^, with higher exposure over North, East, and South Central China, and lower exposure in the GBA, South West, and North West China (Table 1 and Figure S5 in Supporting Information S1). PM_2.5_ exposure is highest across the provinces of Hebei, Beijing, Shandong, Henan, and Anhui in North and East China. The National Air Quality Target (35 μg m^−3^) is achieved in GBA and South West China. The baseline annual disease burden associated with PM_2.5_ exposure in China is estimated as 2,143,700 (95UI: 1,916,200−2,382,400) premature mortalities and 4,219 (95UI: 3,412−5,178) DALYs per 100,000 people. Our estimated disease burdens for 2015 are similar to the previous estimate of 2,440,000 (95UI: 2,046,600−2,808,300) premature mortalities from Burnett et al. (2018). The disease burden rate is largest in North, East, and South Central China.

**Table 1:** The Emulated Baseline (2015) Exposure and Disease Burden for Fine Particulate Matter (PM_2.5_, Annual−Mean) and Ozone (O_3_, Maximum 6−Monthly−Mean Daily−Maximum 8−Hour, 6mDM8h) Across Regions in China

Baseline (2015)	China	GBA	North China	North east China	East China	South central China	South west China	North west China
Annual− mean PM_2.5_ exposure (μg m^−3^)	47.4	26.8	62.3	42.4	47.0	45.3	29.4	37.6
6mDM8h O_3_ exposure (ppb)	43.8	35.7	48.5	41.8	42.8	41.9	41.5	46.2
MORT PM_2.5_ (deaths yr^−1^)	2,143,700 (1,916,200–2,382,400)	66,600 (59,300–74,300)	335,700 (300,800–372,200)	158,200 (141,300–175,900)	651,100 (582,300–723,200)	593,600 (530,600–659,600)	258,200 (230,200–287,800)	147,000 (131,100–163,700)
MORT O_3_ (deaths yr^−1^)	46,000 (31,800–64,900)	1,300 (900–1,900)	8,300 (5,800–11,700)	3,300 (2,300–4,700)	15,300 (10,600–21,500)	10,900 (7,500–15,300)	4,900 (3,400–7,000)	3,200 (2,200–4,600)
DALYs rate PM_2.5_ (DALYs 100,000 people^−1^ yr^−1^)	4,219 (3,412–5,178)	4,318 (3,490–5,304)	5,651 (4,591–6,906)	4,768 (3,861–5,846)	5,431 (4,407–6,645)	5,330 (4,323–6,524)	3,574 (2,885–4,396)	4,231 (3,420–5,197)
DALYs rate O_3_ (DALYs 100,000 people^−1^ yr^−1^)	68 (44–99)	51 (33–74)	112 (73–161)	70 (45–101)	72 (47–104)	64 (41–93)	40 (26–58)	84 (54–122)

*Note.* The disease burden is given by the annual number of premature mortalities (MORT) and the annual rate of disability−adjusted life years (DALYs) per 100,000 people.

Baseline O_3_ exposure in China is 43.8 ppb, with higher exposure over North, North West, and East China, and lower exposure in the GBA, North East, and South Central China (Table 1 and Figure S5 in Supporting Information S1). O_3_ exposure is highest across the provinces of Hubei, Beijing, and Shandong. The National Air Quality Target (80 ppb) and the World Health Organization (WHO) guideline (50 ppb) are achieved in all regions at the baseline (World Health Organization, 2021). The baseline annual disease burden associated with O_3_ exposure in China is estimated as 46,000 (95UI: 31,800−64,900) premature mortalities and 68 (95UI: 44–99) DALYs per 100,000 people. The disease burden rate is largest in North, North West, and East China.

### 3.2 Impact of Changes in Individual Emission Sectors on PM_2.5_ and O_3_ Exposure and Disease Burden

**Figure 2:**
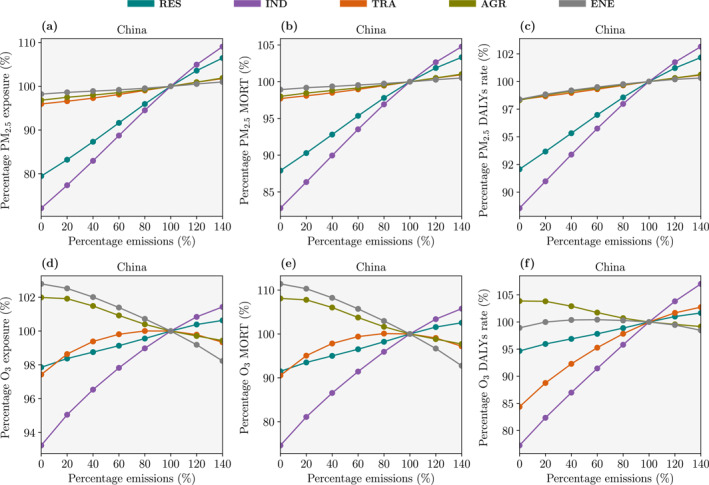
The impact of emission changes in China for 2015 on (a) fine particulate matter (PM_2.5_, annual−mean) exposure, (b) annual premature mortalities (MORT) from PM_2.5_ exposure, (c) annual rate of disability−adjusted life years per 100,000 people from PM_2.5_ exposure, (d) ozone (O_3_, maximum 6−monthly−mean daily−maximum 8−hour, 6mDM8h) exposure, (e) annual MORT from O_3_ exposure, and (f) annual rate of DALYs per 100,000 people from O_3_ exposure. Exposure, MORT, and rate of DALYs are shown relative to a simulation with baseline (100%) emissions. The emission sectors are residential, industry, land transport, agriculture, and power generation.

PM_2.5_ exposure decreases approximately linearly from emission reductions in a single sector (Figure 2a). Completely removing IND emissions decreases national PM_2.5_ exposure by 28% and regional PM_2.5_ exposure by 23%–31%. Under this scenario, the National Air Quality Target (35 μg m^−3^) is achieved in all regions except North China (Figures S7−S13 in Supporting Information S1). Completely removing RES emissions decreases national PM_2.5_ exposure by 21% and regional PM_2.5_ exposure by 8%–28%. Removing IND or RES emissions results in similar reductions in regional PM_2.5_ exposure, except in the GBA where reducing IND emissions provides larger reductions in PM_2.5_ exposure. The impacts on PM_2.5_ exposure from individual emission sector changes are then of decreasing size from TRA, AGR, and ENE emissions.

The sectoral contributions to PM_2.5_ concentrations of 28% from industry, 21% from RES, and 4% from TRA emissions are similar to those from a multi−model study, which found contributions of 30% from industry, 26% from RES, and 7% from TRA emissions (Reddington et al., 2019). We find smaller contributions from AGR (3%) and ENE (2%) emissions compared to the multi−model contribution estimates of 16% from AGR and 14% from ENE emissions (Reddington et al., 2019). In our study, there are larger contributions from other sources (42%, compared to 7% in the multi−model estimates) (Reddington et al., 2019), such as from other anthropogenic emissions inside China, anthropogenic emissions outside China, and natural emissions.

The fractional reductions in PM_2.5_ disease burden (Figure 2b) are smaller than the fractional reductions in PM_2.5_ exposure (Figure 2a), due to the non−linear exposure−outcome association. Completely removing IND emissions decreases the national number of premature mortalities from PM_2.5_ exposure by 17%, avoiding 369,100 (95UI: 334,300–404,800) deaths, and decreases the rate of DALYs by 11%. Completely removing RES emissions decreases the national number of premature mortalities from PM_2.5_ exposure by 12%, and decreases the rate of DALYs by 8%. Removing either IND or RES emissions can decrease the regional number of premature mortalities by 5%–20% and the rate of DALYs by 6%–19%.

O_3_ exposure changes non−linearly with changes in emissions from a single sector (Figure 2d). This non−linear response is stronger for some sectors (e.g., TRA) and in some regions (e.g., North China, Figures S7−S13 in Supporting Information S1). Completely removing either IND or RES emissions consistently decreases O_3_ exposure. Removing IND emissions decreases national O_3_ exposure by 7% and regional O_3_ exposure by 3%–10%. Removing RES emissions decreases national O_3_ exposure by 2% and regional O_3_ exposure by 0%–4%. However, individually removing ENE, TRA, or AGR emissions can increase O_3_ exposure.

The fractional changes in the O_3_ disease burden (Figure 2e) are larger than the fractional changes in O_3_ exposure (Figure 2d), due to the high counterfactual exposure level of no excess risk. Completely removing IND emissions decreases the national number of premature mortalities from O_3_ exposure by 25%, avoiding 11,700 (95UI: 8,100−16,400) deaths, and decreases the rate of DALYs by 23%. Completely removing RES emissions decreases the national number of premature mortalities from O_3_ exposure by 9%, avoiding 3,900 (95UI: 2,700−5,500) deaths, and decreases the rate of DALYs by 5%. Although removing TRA emissions decreases the national number of premature mortalities from O_3_ exposure by 9% avoiding 4,400 (95UI: 3,000–6,100) deaths, the number of premature mortalities in the GBA increases by 21%, an additional 300 (95UI: 200–400) deaths locally. Removing ENE emissions increases the national number of premature mortalities from O_3_ exposure by 11%, whilst removing AGR emissions increases the national number of premature mortalities from O_3_ exposure by 8%.

### 3.3 Impact of Changes in Multiple Emission Sectors on PM_2.5_ and O_3_ Exposure and Disease Burden

**Figure 3:**
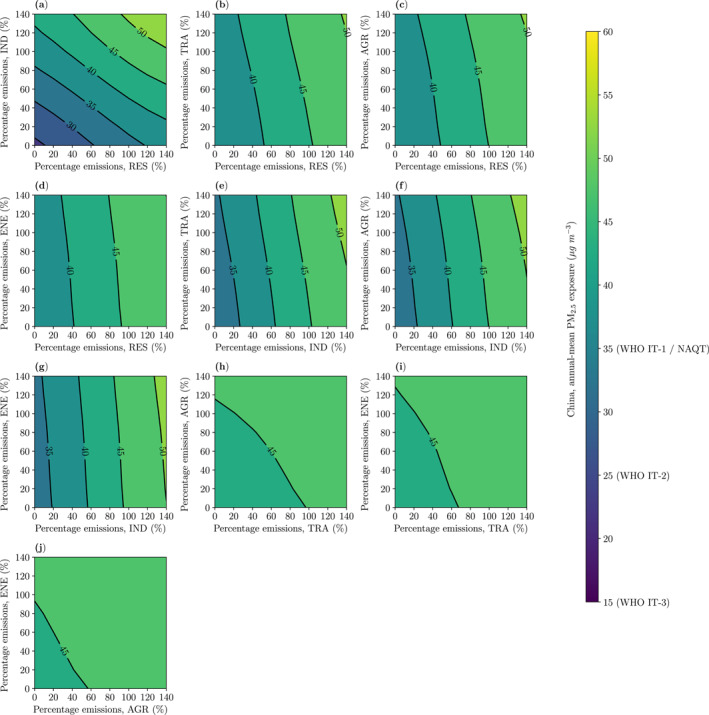
The impact of variations in two emission sectors on fine particulate matter (PM_2.5_, annual−mean) exposure in China for 2015 from (a) residential (RES) and industry (IND), (b) RES and land transport (TRA), (c) RES and agriculture (AGR), (d) RES and power generation (ENE), (e) IND and TRA, (f) IND and AGR, (g) IND and ENE, (h) TRA and AGR, (i) TRA and ENE, and (j) AGR and ENE emissions. Air quality targets shown for the World Health Organization's (WHO) Interim Target 1 (IT−1, 35 μg m^−3^), Interim Target 2 (IT−2, 25 μg m^−3^), Interim Target 3 (IT−3, 15 μg m^−3^), and China's National Air Quality Target (35 μg m^−3^).

Removing RES and IND emissions together decreases national PM_2.5_ exposure by 48% (Figure 3) and regional PM_2.5_ exposure by 37%–53% (Figures S14−S20 in Supporting Information S1). These emission reductions attain the WHO Interim Target 2 (25 μg m^−3^) in all regions except North and East China, and attain the WHO Interim Target 3 (15 μg m^−3^) in the GBA. Removing IND emissions with either AGR, TRA, or ENE emissions attains the National Air Quality Target (35 μg m^−3^) in China (Figure 3). Removing TRA, AGR, and ENE emissions, without reducing RES and IND emissions, decreases national PM_2.5_ exposure by 9%.

Removing emissions from all five sectors together decreases national PM_2.5_ exposure by 57% and regional PM_2.5_ exposure by 52%–61%. These emission reductions attain the WHO Interim Target 2 (25 μg m^−3^) in all regions except North China, but only attain the WHO Interim Target 3 (15 μg m^−3^) in the GBA. The WHO Annual Guideline (5 μg m^−3^) is not achieved in any region. Under this scenario, PM_2.5_ concentrations result from other anthropogenic emissions inside China including shipping, aviation, and agricultural fires, from anthropogenic emissions outside China, and from natural emission sources such as vegetation fires, dust, sea spray, and secondary organic aerosols from biogenic volatile organic compounds (VOC). Previous studies have estimated the contributions to PM_2.5_ concentrations in China from dust were 2%–10% and were higher in North West China (Shi et al., 2017; Yang et al., 2011). Open biomass burning was estimated to contribute 1%–8% of PM_2.5_ concentrations across China (Reddington et al., 2019), with higher contributions of up to 29% in South Central China (Reddington et al., 2019; Shi et al., 2017). Biogenic SOA has been estimated to contribute 2%–8% of PM_2.5_ concentrations (Hu et al., 2017; Shi et al., 2017). Long−range transport of PM_2.5_ concentrations from anthropogenic emissions outside China was estimated to contribute up to 3% of PM_2.5_ concentrations inside China (Liu et al., 2020). Anthropogenic emissions from shipping were estimated to contribute up to 3% (Chen et al., 2019; Dasadhikari et al., 2019; Reddington et al., 2019) and aviation 1% (Dasadhikari et al., 2019; Zhang et al., 2017). Emissions from sea salt have been estimated to contribute 1% of PM_2.5_ concentrations (Shi et al., 2017).

**Figure 4:**
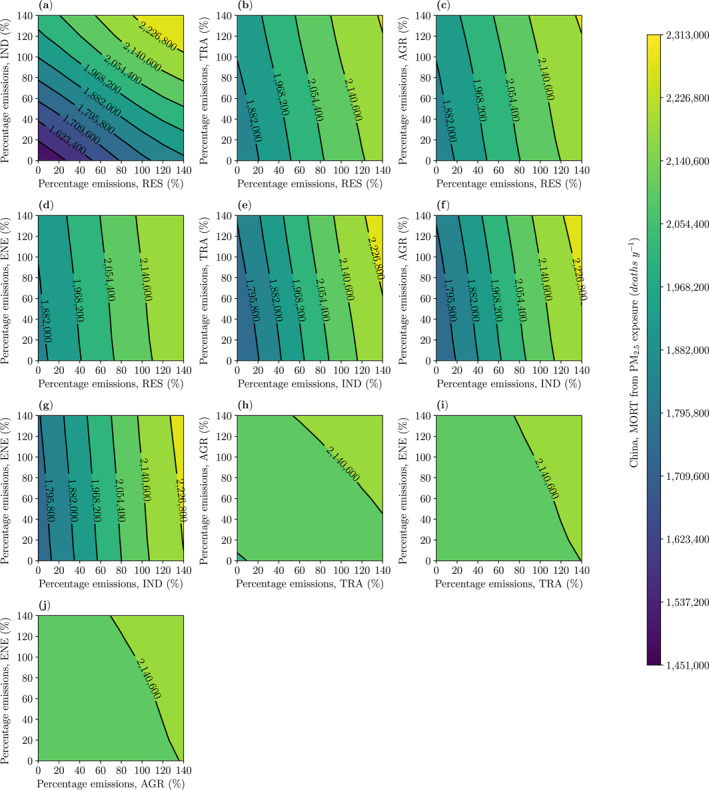
The impact of variations in two emission sectors on the disease burden (premature mortalities, MORT, per year) from fine particulate matter (PM_2.5_, annual−mean) exposure for China from (a) residential (RES) and industry (IND), (b) RES and land transport (TRA), (c) RES and agriculture (AGR), (d) RES and power generation (ENE), (e) IND and TRA, (f) IND and AGR, (g) IND and ENE, (h) TRA and AGR, (i) TRA and ENE, and (j) AGR and ENE emissions.

Removing RES and IND emissions together decreases the national number of premature mortalities from PM_2.5_ exposure by 32%, avoiding 691,800 (95UI: 625,100–760,400) deaths, and decreases the rate of DALYs by 21% (Figure 4 and S22 in Supporting Information S1). Removing TRA, AGR, and ENE emissions together decreases the national number of premature mortalities from PM_2.5_ exposure by 5%. Removing emissions from all five sectors together decreases the national number of premature mortalities from PM_2.5_ exposure by 40%, avoiding 858,800 (95UI: 774,900–945,400) deaths, and decreases the rate of DALYs by 27%.

These findings highlight that substantial public health benefits can be achieved by emission reductions, and the majority of these benefits due to reductions in IND and RES emissions. However, even after removing emissions from these five sectors, approximately half of the disease burden associated with PM_2.5_ exposure remains, due to other sources of air pollution.

The largest reductions in O_3_ exposure occur when removing IND emissions with either TRA or RES emissions (Figure S22 in Supporting Information S1). Removing IND and TRA emissions decreases national O_3_ exposure by 14% and regional O_3_ exposure by 8%–22% (Figures S23−S29 in Supporting Information S1), reducing the disease burden by 46% and the rate of DALYs by 41% (Figures S30 and S31 in Supporting Information S1). Removing IND and RES emissions decreases national O_3_ exposure by 10% and regional O_3_ exposure by 5%–16% reducing the annual number of premature mortalities by 36%. However, some combinations of reductions in ENE, AGR, and TRA emissions can increase O_3_ exposure and the associated disease burden in some regions.

These findings highlight the complex dependencies between the chemical production of O_3_ and precursors of O_3_, especially nitrogen oxides (NO_X_) and VOC emissions. The ENE and TRA sectors have large NO_X_ emissions but smaller VOC emissions, while the IND sector has large emissions of both NO_X_ and VOC (Zheng et al., 2018). As some urban areas in East China are VOC−limited (Jin & Holloway, 2015; Wang et al., 2017), reducing NO_X_ emissions from the ENE and TRA sectors can increase O_3_ exposure (Li et al., 2021).

The largest public health benefits come from reductions in PM_2.5_ exposure, as the PM_2.5_ disease burden is two orders of magnitude larger the O_3_ disease burden. The largest public health benefits also come from reductions in IND and RES emissions, as these sectors dominate PM_2.5_ exposure and are the only sectors which consistently decrease O_3_ exposure.

### 3.4 Emission Configurations That Meet Air Quality Targets

**Figure 5:**
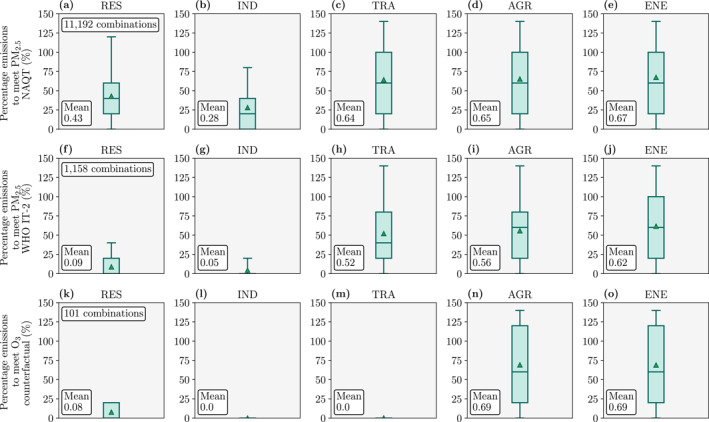
Emission configurations that meet air quality targets in China from the baseline in 2015. Targets are (a–e) the National Air Quality Target (35 μg m^−3^) for ambient fine particulate matter (PM_2.5_, annual−mean) concentrations (f–j) the World Health Organization Interim Target 2 (IT−2, 25 μg m^−3^) for PM_2.5_ concentrations, and (k–o) the counterfactual exposure level of no excess risk for ozone (O_3_, 35.7 ppb) concentrations. Boxplots per sector from (a, f, and k) residential (RES), (b, g, and l) industrial (IND), (c, h, and m) land transport (TRA), (d, i, and n) agricultural (AGR), and (e, j, and o) power generation (ENE) emissions.

There are 11,192 emission configurations that meet the National Air Quality Target (35 μg m^−3^) nationally for PM_2.5_ concentrations, requiring mean emission reductions of 72% in IND, 57% in RES, 36% in TRA, 35% in AGR, and 33% in ENE emissions (Figure 5). The WHO Interim Target 2 (25 μg m^−3^) can be attained nationally for PM_2.5_ concentrations via 1,158 emission configurations, requiring 95% reductions in IND and RES emissions. The GBA is the only region where the WHO Interim Target 3 (15 μg m^−3^) can be attained, requiring stringent reductions in IND emissions (Figure S32 in Supporting Information S1). The WHO Air Quality Guideline (5 μg m^−3^) cannot be attained in any region from reductions in these five emission sectors alone.

For O_3_ concentrations, the National Air Quality Target (80 ppb) and the WHO guideline (50 ppb) are already attained at the baseline. There are 101 emission configurations that can reach the counterfactual exposure level of no excess risk (35.7 ppb) for O_3_ concentrations, all of which require the near removal of IND, TRA, and RES emissions. Reaching this level would remove the health impacts associated with O_3_ exposure. This attainment is possible in all regions except North and East China (Figure S32 in Supporting Information S1).

## 4 Conclusion

We developed emulators to predict annual mean PM_2.5_ and O_3_ concentrations and associated public health impacts in China. Our analysis provides a first estimate of how air pollution exposure and associated disease burden in China vary for different emission reductions across five major emission sectors (IND, RES, TRA, AGR, and ENE). The emulators predicted 99.9% of the variance in PM_2.5_ and O_3_ concentrations for a given input configuration of emissions. We found that PM_2.5_ exposure was most sensitive to IND and RES emissions, confirming previous studies (Reddington et al., 2019). Complete removal of IND emissions would attain the National Air Quality Target (35 μg m^−3^) in all regions except North China. The National Air Quality Target can be attained nationally with emission reductions of 72% in IND, 57% in RES, 36% in TRA, 35% in AGR, and 33% in ENE emissions. Removing RES and IND emissions completely, decreases national PM_2.5_ exposure by 48% (to 24.0 μg m^−3^). However, removing emissions from the five sectors in China does not enable the attainment of the WHO Annual Guideline (5 μg m^−3^) due to the remaining emissions from shipping, aviation, and agricultural fires, emissions from outside China, and from natural emission sources including forest fires, dust, and biogenics.

Emulators have broad potential in air quality research. Future work could study other regions, countries, emission sources, and could further split emission inputs by species, sub−sectors, and time−of−day. Here, we chose to apply the same emission changes over all species within each sector due to computational constraints. For five inputs, 55 annual high–resolution WRFChem simulations were required for the training and testing data (Loeppky et al., 2009). If the emulators split the emissions by pollutant, then the computational burden would have increased by up to a factor of 10. Our work was conducted for one meteorological year (2015). Previous work found that the air quality impacts from meteorological changes were smaller than those from emission changes (Ding et al., 2019; Silver, Conibear, et al., 2020; Silver, He et al., 2020). However, future work should account for and explore variations in meteorology, including seasonal and inter−annual variations. To further understand how China can achieve the WHO Annual Guideline for PM_2.5_ exposure (5 μg m^−3^), future work is needed exploring the relative contributions of other anthropogenic emission sources, emissions outside China and natural emissions, which may increase under climate change (Carslaw et al., 2010). Our work highlights the challenges facing China as it attempts to further reduce PM_2.5_ exposure and improve public health.

## Conflict of Interest

The authors declare no conflicts of interest relevant to this study.

## Supporting information

Supporting Information S1

## Data Availability

Code to setup and run WRFChem (using WRFotron version 2.0) is available through Conibear and Knote (2020). Emulator code and data is available through Conibear. (2021). The trained emulators per grid cell in China that support the findings of this study are available in Conibear et al. (2022b).
