# Supplementary material for: Sensitivity of Air Pollution Exposure and Disease Burden to Emission Changes in China Using Machine Learning Emulation
Source: Geohealth. 2022 Jun 1;6(6):e2021GH000570. doi: 10.1029/2021GH000570 (PMC9207901; doi:10.1029/2021GH000570)
Supplement: Supplementary file 1 — Supporting Information S1 [file GH2-6-e2021GH000570-s001.pdf]

Supporting information for

Sensitivity of Air Pollution Exposure and Disease Burden to Emission Changes in China using Machine Learning Emulation

Luke Conibear[*,1], Carly L. Reddington[1], Ben J. Silver[1], Ying Chen[2], Christoph Knote[3], Stephen R. Arnold[1], and Dominick V. Spracklen[1]

[1] Institute for Climate and Atmospheric Science, School of Earth and Environment, University of Leeds, Leeds, UK

[2] College of Engineering, Mathematics and Physical Sciences, University of Exeter, UK

[3] Faculty of Medicine, University of Augsburg, Germany

* Corresponding author: Luke Conibear (L.A.Conibear@leeds.ac.uk)

Contents

Table S1: Scaling factors for each anthropogenic emission sector of the training runs. Applied to all species within the sector. Sectors are residential (RES), industry (IND), land transport (TRA), agriculture (AGR), and power generation (ENE).

Table S2: Scaling factors for each anthropogenic emission sector of the testing runs. Applied to all species within the sector. Sectors are residential (RES), industry (IND), land transport (TRA), agriculture (AGR), and power generation (ENE).

Figure S1: Anthropogenic emissions of fine particulate matter ($PM_{2.5}$) in 2015 across China from the (a) residential (RES), (b), industrial (IND), (c) land transport (TRA), and (d) power generation (ENE) sectors.

Figure S2: Anthropogenic emissions of ammonia (NH_3) in 2015 across China from the (a) residential (RES), (b), industrial (IND), (c) land transport (TRA), and (d) agricultural (AGR) sectors.

Figure S3: Anthropogenic emissions of nitrogen oxides (NO_X) in 2015 across China from the (a) residential (RES), (b), industrial (IND), (c) land transport (TRA), and (d) power generation (ENE) sectors.

Figure S4: Anthropogenic emissions of non−methane volatile organic compounds (VOC) in 2015 across China from the (a) residential (RES), (b), industrial (IND), (c) land transport (TRA), and (d) power generation (ENE) sectors.

Figure S5: Evaluation of unscaled and scaled baseline simulator air quality concentrations. Regional evaluation metrics are normalised mean bias factor (NMBF) grouped by prefecture if available, otherwise by province for (a) for fine particulate matter ($PM_{2.5}$, annual−mean) and (d) ozone (O_3, maximum 6−monthly−mean daily−maximum 8−hour, 6mDM8h). Unscaled baseline concentrations for (b) $PM_{2.5}$ (NMBF = −0.05, normalised absolute error factor, NMAEF, = 0.18) and (e) O_3 (NMBF = 0.39, NMAEF = 0.40). Scaled baseline concentrations for (c) $PM_{2.5}$ (NMBF = 0.02, NMAEF, = 0.10) and (f) O_3 (NMBF = 0.03, NMAEF = 0.11).

Figure S6: Directed acyclic graph of the emulator workflow. Information in brackets represents information that is specific to this work. The emulator inputs were anthropogenic emissions from the residential (RES), industrial (IND), land transport (TRA), agricultural (AGR), and power generation (ENE) sectors. The emulator outputs were annual−mean fine particulate matter ($PM_{2.5}$) concentrations and maximum 6−monthly−mean daily−maximum 8−hour (6mDM8h) ozone (O_3) concentrations. The simulator was WRFChem (Weather Research and Forecasting model online–coupled with Chemistry), with anthropogenic emissions from MEIC (Multi–resolution Emission Inventory for China), gas phase chemistry from MOZART (Model for Ozone and Related Chemical Tracers), and aerosol chemistry and physics from MOSAIC (Model for Simulating Aerosol Interactions and Chemistry). The health impact assessment used exposure−outcome associations from the GEMM (Global Exposure Mortality Model) for non−accidental mortality (non−communicable disease, NCD, plus lower respiratory infections, LRI) for $PM_{2.5}$ exposure and from the Global Burden of Diseases, Injuries, and Risk Factors Study (GBD) from 2017 for O_3 exposure. See the Methods for more information on each step.

Figure S7: The fractional impact of individual emission changes in the Guangdong−Hong Kong−Macau Greater Bay Area (GBA) on (a) fine particulate matter ($PM_{2.5}$, annual−mean) exposure, (b) annual premature mortalities (MORT) from $PM_{2.5}$ exposure, (c) annual rate of disability−adjusted life years (DALYs) per 100,000 people from

$PM_{2.5}$ exposure, (d) ozone (O_3, maximum 6−monthly−mean daily−maximum 8−hour, 6mDM8h) exposure, (e) annual MORT from O_3 exposure, and (f) annual rate of DALYs per 100,000 people from O_3 exposure. The five emission sectors are residential (RES), industry (IND), land transport (TRA), agriculture (AGR), and power generation (ENE).

Figure S8: The fractional impact of individual emission changes in North China on (a) fine particulate matter ($PM_{2.5}$, annual−mean) exposure, (b) annual premature mortalities (MORT) from $PM_{2.5}$ exposure, (c) annual rate of disability−adjusted life years (DALYs) per 100,000 people from $PM_{2.5}$ exposure, (d) ozone (O_3, maximum 6−monthly−mean daily−maximum 8−hour, 6mDM8h) exposure, (e) annual MORT from O_3 exposure, and (f) annual rate of DALYs per 100,000 people from O_3 exposure. The five emission sectors are residential (RES), industry (IND), land transport (TRA), agriculture (AGR), and power generation (ENE).

Figure S9: The fractional impact of individual emission changes in North East China on (a) fine particulate matter ($PM_{2.5}$, annual−mean) exposure, (b) annual premature mortalities (MORT) from $PM_{2.5}$ exposure, (c) annual rate of disability−adjusted life years (DALYs) per 100,000 people from $PM_{2.5}$ exposure, (d) ozone (O_3, maximum 6−monthly−mean daily−maximum 8−hour, 6mDM8h) exposure, (e) annual MORT from O_3 exposure, and (f) annual rate of DALYs per 100,000 people from O_3 exposure. The five emission sectors are residential (RES), industry (IND), land transport (TRA), agriculture (AGR), and power generation (ENE).

Figure S10: The fractional impact of individual emission changes in East China on (a) fine particulate matter ($PM_{2.5}$, annual−mean) exposure, (b) annual premature mortalities (MORT) from $PM_{2.5}$ exposure, (c) annual rate of disability−adjusted life years (DALYs) per 100,000 people from $PM_{2.5}$ exposure, (d) ozone (O_3, maximum 6−monthly−mean daily−maximum 8−hour, 6mDM8h) exposure, (e) annual MORT from O_3 exposure, and (f) annual rate of DALYs per 100,000 people from O_3 exposure. The five emission sectors are residential (RES), industry (IND), land transport (TRA), agriculture (AGR), and power generation (ENE).

Figure S11: The fractional impact of individual emission changes in South Central China on (a) fine particulate matter ($PM_{2.5}$, annual−mean) exposure, (b) annual premature mortalities (MORT) from $PM_{2.5}$ exposure, (c) annual rate of disability−adjusted life years (DALYs) per 100,000 people from $PM_{2.5}$ exposure, (d) ozone (O_3, maximum 6−monthly−mean daily−maximum 8−hour, 6mDM8h) exposure, (e) annual MORT from O_3 exposure, and (f) annual rate of DALYs per 100,000 people from O_3 exposure. The five emission sectors are residential (RES), industry (IND), land transport (TRA), agriculture (AGR), and power generation (ENE).

Figure S12: The fractional impact of individual emission changes in South West China on (a) fine particulate matter ($PM_{2.5}$, annual−mean) exposure, (b) annual premature mortalities (MORT) from $PM_{2.5}$ exposure, (c) annual rate of disability−adjusted life years (DALYs) per 100,000 people from $PM_{2.5}$ exposure, (d) ozone (O_3, maximum 6−monthly−mean daily−maximum 8−hour, 6mDM8h) exposure, (e) annual MORT from O_3 exposure, and (f) annual rate of DALYs per 100,000 people from O_3 exposure. The five emission sectors are residential (RES), industry (IND), land transport (TRA), agriculture (AGR), and power generation (ENE).

Figure S13: The fractional impact of individual emission changes in North West China on (a) fine particulate matter ($PM_{2.5}$, annual−mean) exposure, (b) annual premature mortalities (MORT) from $PM_{2.5}$ exposure, (c) annual rate of disability−adjusted life years (DALYs) per 100,000 people from $PM_{2.5}$ exposure, (d) ozone (O_3, maximum 6−monthly−mean daily−maximum 8−hour, 6mDM8h) exposure, (e) annual MORT from O_3 exposure, and (f) annual rate of DALYs per 100,000 people from O_3 exposure. The five emission sectors are residential (RES), industry (IND), land transport (TRA), agriculture (AGR), and power generation (ENE).

Figure S14: The impact of variations in two emission sectors on fine particulate matter ($PM_{2.5}$, annual−mean) exposure for Guangdong−Hong Kong−Macau Greater Bay Area (GBA) from (a) residential (RES) and industry (IND), (b) RES and land transport (TRA), (c) RES and agriculture (AGR), (d) RES and power generation (ENE), (e) IND and TRA, (f) IND and AGR, (g) IND and ENE, (h) TRA and AGR, (i) TRA and ENE, and (j) AGR and ENE emissions. Air quality targets shown for the World Health Organization's (WHO) Air Quality Guideline (AQG, 5 $\mu g\ m^{-3}$), Interim Target 1 (IT−1, 35 $\mu g\ m^{-3}$), Interim Target 2 (IT−2, 25 $\mu g\ m^{-3}$), Interim Target 3 (IT−3, 15 $\mu g\ m^{-3}$), Interim Target 4 (IT−4, 10 $\mu g\ m^{-3}$), and China's National Air Quality Target (NAQT, 35 $\mu g\ m^{-3}$).

Figure S15: The impact of variations in two emission sectors on fine particulate matter ($PM_{2.5}$, annual−mean) exposure for North China from (a) residential (RES) and industry (IND), (b) RES and land transport (TRA), (c) RES and agriculture (AGR), (d) RES and power generation (ENE), (e) IND and TRA, (f) IND and AGR, (g) IND and ENE, (h) TRA and AGR, (i) TRA and ENE, and (j) AGR and ENE emissions. Air quality targets shown for the World Health Organization's (WHO) Air Quality Guideline (AQG, 5 $\mu g\ m^{-3}$), Interim Target 1 (IT−1, 35 $\mu g\ m^{-3}$), Interim Target 2 (IT−2, 25 $\mu g\ m^{-3}$), Interim Target 3 (IT−3, 15 $\mu g\ m^{-3}$), Interim Target 4 (IT−4, 10 $\mu g\ m^{-3}$), and China's National Air Quality Target (NAQT, 35 $\mu g\ m^{-3}$).

Figure S16: The impact of variations in two emission sectors on fine particulate matter ($PM_{2.5}$, annual−mean) exposure for North East China from (a) residential (RES) and industry (IND), (b) RES and land transport (TRA), (c) RES and agriculture (AGR), (d) RES and power generation (ENE), (e) IND and TRA, (f) IND and AGR, (g) IND and ENE, (h) TRA and AGR, (i) TRA and ENE, and (j) AGR and ENE emissions. Air quality targets shown for the World Health Organization's (WHO) Air Quality Guideline (AQG, 5 $\mu g\ m^{-3}$), Interim Target 1 (IT−1, 35 $\mu g\ m^{-3}$), Interim Target 2 (IT−2, 25 $\mu g\ m^{-3}$), Interim Target 3 (IT−3, 15 $\mu g\ m^{-3}$), Interim Target 4 (IT−4, 10 $\mu g\ m^{-3}$), and China's National Air Quality Target (NAQT, 35 $\mu g\ m^{-3}$).

Figure S17: The impact of variations in two emission sectors on fine particulate matter ($PM_{2.5}$, annual−mean) exposure for East China from (a) residential (RES) and industry (IND), (b) RES and land transport (TRA), (c) RES and agriculture (AGR), (d) RES and power generation (ENE), (e) IND and TRA, (f) IND and AGR, (g) IND and ENE, (h) TRA and AGR, (i) TRA and ENE, and (j) AGR and ENE emissions. Air quality targets shown for the World Health Organization's (WHO) Air Quality Guideline (AQG, 5 $\mu g\ m^{-3}$), Interim Target 1 (IT−1, 35 $\mu g\ m^{-3}$), Interim Target 2 (IT−2, 25 $\mu g\ m^{-3}$), Interim Target 3 (IT−3, 15 $\mu g\ m^{-3}$), Interim Target 4 (IT−4, 10 $\mu g\ m^{-3}$), and China's National Air Quality Target (NAQT, 35 $\mu g\ m^{-3}$).

Figure S18: The impact of variations in two emission sectors on fine particulate matter ($PM_{2.5}$, annual−mean) exposure for South Central China from (a) residential (RES) and industry (IND), (b) RES and land transport (TRA), (c) RES and agriculture (AGR), (d) RES and power generation (ENE), (e) IND and TRA, (f) IND and AGR, (g) IND and ENE, (h) TRA and AGR, (i) TRA and ENE, and (j) AGR and ENE emissions. Air quality targets shown for the World Health Organization's (WHO) Air Quality Guideline (AQG, 5 $\mu g\ m^{-3}$), Interim Target 1 (IT−1, 35 $\mu g\ m^{-3}$), Interim Target 2 (IT−2, 25 $\mu g\ m^{-3}$), Interim Target 3 (IT−3, 15 $\mu g\ m^{-3}$), Interim Target 4 (IT−4, 10 $\mu g\ m^{-3}$), and China's National Air Quality Target (NAQT, 35 $\mu g\ m^{-3}$).

Figure S19: The impact of variations in two emission sectors on fine particulate matter ($PM_{2.5}$, annual−mean) exposure for South West China from (a) residential (RES) and industry (IND), (b) RES and land transport (TRA), (c) RES and agriculture (AGR), (d) RES and power generation (ENE), (e) IND and TRA, (f) IND and AGR, (g) IND and ENE, (h) TRA and AGR, (i) TRA and ENE, and (j) AGR and ENE emissions. Air quality targets shown for the World Health Organization's (WHO) Air Quality Guideline (AQG, 5 $\mu g\ m^{-3}$), Interim Target 1 (IT−1, 35 $\mu g\ m^{-3}$), Interim Target 2 (IT−2, 25 $\mu g\ m^{-3}$), Interim Target 3 (IT−3, 15 $\mu g\ m^{-3}$), Interim Target 4 (IT−4, 10 $\mu g\ m^{-3}$), and China's National Air Quality Target (NAQT, 35 $\mu g\ m^{-3}$).

Figure S20: The impact of variations in two emission sectors on fine particulate matter ($PM_{2.5}$, annual−mean) exposure for North West China from (a) residential (RES) and industry (IND), (b) RES and land transport (TRA), (c) RES and agriculture (AGR), (d) RES and power generation (ENE), (e) IND and TRA, (f) IND and AGR, (g) IND and ENE, (h) TRA and AGR, (i) TRA and ENE, and (j) AGR and ENE emissions. Air quality targets shown for the World Health Organization's (WHO) Air Quality Guideline (AQG, 5 $\mu g\ m^{-3}$), Interim Target 1 (IT−1, 35 $\mu g\ m^{-3}$), Interim Target 2 (IT−2, 25 $\mu g\ m^{-3}$), Interim Target 3 (IT−3, 15 $\mu g\ m^{-3}$), Interim Target 4 (IT−4, 10 $\mu g\ m^{-3}$), and China's National Air Quality Target (NAQT, 35 $\mu g\ m^{-3}$).

Figure S21: The impact of variations in two emission sectors on the disease burden (rate of disability−adjusted life years, DALYs, per 100,000 people per year) from fine particulate matter ($PM_{2.5}$, annual−mean) exposure for China from (a) residential (RES) and industry (IND), (b) RES and land transport (TRA), (c) RES and agriculture (AGR), (d) RES and power generation (ENE), (e) IND and TRA, (f) IND and AGR, (g) IND and ENE, (h) TRA and AGR, (i) TRA and ENE, and (j) AGR and ENE emissions.

Figure S22: The impact of variations in two emission sectors on ozone (O_3, maximum 6−monthly−mean daily−maximum 8−hour, 6mDM8h) exposure in China for 2015 from (a) residential (RES) and industry (IND), (b) RES and land transport (TRA), (c) RES and agriculture (AGR), (d) RES and power generation (ENE), (e) IND and TRA, (f) IND and AGR, (g) IND and ENE, (h) TRA and AGR, (i) TRA and ENE, and (j) AGR and ENE emissions.

Figure S23: The impact of variations in two emission sectors on ozone (O_3, maximum 6−monthly−mean daily−maximum 8−hour, 6mDM8h) exposure for Guangdong−Hong Kong−Macau Greater Bay Area (GBA) from (a) residential (RES) and industry (IND), (b) RES and land transport (TRA), (c) RES and agriculture (AGR), (d) RES and power generation (ENE), (e) IND and TRA, (f) IND and AGR, (g) IND and ENE, (h) TRA and AGR, (i) TRA and ENE, and (j) AGR and ENE emissions.

Figure S24: The impact of variations in two emission sectors on ozone (O_3, maximum 6−monthly−mean daily−maximum 8−hour, 6mDM8h) exposure for North China from (a) residential (RES) and industry (IND), (b) RES and land transport (TRA), (c) RES and agriculture (AGR), (d) RES and power generation (ENE), (e) IND and TRA, (f) IND and AGR, (g) IND and ENE, (h) TRA and AGR, (i) TRA and ENE, and (j) AGR and ENE emissions.

Figure S25: The impact of variations in two emission sectors on ozone (O_3, maximum 6−monthly−mean daily−maximum 8−hour, 6mDM8h) exposure for North East China from (a) residential (RES) and industry (IND), (b) RES and land transport (TRA), (c) RES and agriculture (AGR), (d) RES and power generation (ENE), (e) IND and TRA, (f) IND and AGR, (g) IND and ENE, (h) TRA and AGR, (i) TRA and ENE, and (j) AGR and ENE emissions. Air quality targets shown for the World Health Organization's (WHO) Air Quality Guideline (AQG, 50 ppb).

Figure S26: The impact of variations in two emission sectors on ozone (O_3, maximum 6−monthly−mean daily−maximum 8−hour, 6mDM8h) exposure for East China from (a) residential (RES) and industry (IND), (b) RES and land transport (TRA), (c) RES and agriculture (AGR), (d) RES and power generation (ENE), (e) IND and TRA, (f) IND and AGR, (g) IND and ENE, (h) TRA and AGR, (i) TRA and ENE, and (j) AGR and ENE emissions.

Figure S27: The impact of variations in two emission sectors on ozone (O_3, maximum 6−monthly−mean daily−maximum 8−hour, 6mDM8h) exposure for South Central China from (a) residential (RES) and industry (IND), (b) RES and land transport (TRA), (c) RES and agriculture (AGR), (d) RES and power generation (ENE), (e) IND and TRA, (f) IND and AGR, (g) IND and ENE, (h) TRA and AGR, (i) TRA and ENE, and (j) AGR and ENE emissions.

Figure S28: The impact of variations in two emission sectors on ozone (O_3, maximum 6−monthly−mean daily−maximum 8−hour, 6mDM8h) exposure for South West China from (a) residential (RES) and industry (IND), (b) RES and land transport (TRA), (c) RES and agriculture (AGR), (d) RES and power generation (ENE), (e) IND and TRA, (f) IND and AGR, (g) IND and ENE, (h) TRA and AGR, (i) TRA and ENE, and (j) AGR and ENE emissions.

Figure S29: The impact of variations in two emission sectors on ozone (O_3, maximum 6−monthly−mean daily−maximum 8−hour, 6mDM8h) exposure for North West China from (a) residential (RES) and industry (IND), (b) RES and land transport (TRA), (c) RES and agriculture (AGR), (d) RES and power generation (ENE), (e) IND and TRA, (f) IND and AGR, (g) IND and ENE, (h) TRA and AGR, (i) TRA and ENE, and (j) AGR and ENE emissions.

Figure S30: The impact of variations in two emission sectors on the disease burden (premature mortalities, MORT, per year) from ozone (O_3, maximum 6−monthly−mean daily−maximum 8−hour, 6mDM8h) exposure for China from (a) residential (RES) and industry (IND), (b) RES and land transport (TRA), (c) RES and agriculture (AGR), (d) RES and power generation (ENE), (e) IND and TRA, (f) IND and AGR, (g) IND and ENE, (h) TRA and AGR, (i) TRA and ENE, and (j) AGR and ENE emissions.

Figure S31: The impact of variations in two emission sectors on the disease burden (rate of disability−adjusted life years, DALYs, per 100,000 people per year) from ozone (O_3, maximum 6−monthly−mean daily−maximum 8−hour, 6mDM8h) exposure for China from (a) residential (RES) and industry (IND), (b) RES and land transport (TRA), (c) RES and agriculture (AGR), (d) RES and power generation (ENE), (e) IND and TRA, (f) IND and AGR, (g) IND and ENE, (h) TRA and AGR, (i) TRA and ENE, and (j) AGR and ENE emissions.

Figure S32: Mean emission configurations that meet air quality targets regionally across China. The number of emission combinations that meet the air quality targets are given per target and region. The horizontal line at 70% emissions is the average emissions if all combinations meet the air quality target. Targets are the (a) National Air Quality Target (NAQT, 35 $\mu g\ m^{-3}$) for ambient fine particulate matter ($PM_{2.5}$) concentrations, (b) World Health Organization (WHO) Interim Target 2 (IT−2, 25 $\mu g\ m^{-3}$) for $PM_{2.5}$ concentrations, (c) WHO Interim Target 3 (IT−3, 15 $\mu g\ m^{-3}$) for $PM_{2.5}$ concentrations, (d) WHO Air Quality Guideline (AQG, 5 $\mu g\ m^{-3}$) for $PM_{2.5}$ concentrations, and (e) counterfactual exposure level of no excess risk for ozone (O_3) concentrations. Regions are Guangdong−Hong Kong−Macau Greater Bay Area (GBA) and North, North East, East, South Central, South West, and North West China. Sectors are residential (RES), industrial (IND), land transport (TRA), agricultural (AGR), and power generation (ENE) emissions.

Additional Supporting Information (Files uploaded separately)

The trained emulators per grid cell in China that support the findings of this study are available in Conibear et al., (2022).

Table S1: Scaling factors for each anthropogenic emission sector of the training runs. Applied to all species within the sector. Sectors are residential (RES), industry (IND), land transport (TRA), agriculture (AGR), and power generation (ENE).

Run	RES	IND	TRA	AGR	ENE
1	0.232290	0.205070	0.299040	0.973740	0.551840
2	1.121200	1.109800	0.870100	1.222200	1.252000
3	0.798430	1.294600	0.090709	0.754010	0.194200
4	0.344450	1.422400	0.751120	1.240900	1.211300
5	0.625620	1.358900	1.058300	0.046880	0.641960
6	1.317300	1.481600	1.260400	0.172230	1.124200
7	0.322090	1.224400	1.358300	0.815870	0.881020
8	0.504950	1.032500	0.623790	0.346780	0.517600
9	1.081500	0.589920	0.174440	0.903560	0.914420
10	1.047700	0.852830	1.021800	0.662300	0.295090
11	0.200370	0.938770	0.787540	0.840040	0.380760
12	0.568470	1.344100	0.243480	1.023100	1.145800
13	0.921400	1.452800	1.308100	1.072500	0.976740
14	1.057700	0.665280	0.449540	0.899190	0.215160
15	0.748300	0.484550	1.404100	0.514490	1.023300
16	0.110840	1.074500	0.008116	1.272900	0.110040
17	1.159500	1.286100	0.591250	0.747910	0.662940
18	0.672560	0.973790	1.148300	0.490410	0.571260
19	0.644860	0.133860	1.209700	0.544930	1.362400
20	0.017270	0.878860	0.418380	0.623740	0.712510
21	0.377570	1.168500	0.991360	0.587440	1.300400
22	0.771580	1.244500	1.253700	1.353400	1.450900
23	1.372900	0.039672	0.325750	1.316900	0.760170
24	0.877930	0.416510	0.374790	0.383360	0.353590
25	0.590670	0.384670	1.112800	1.148300	1.480700
26	0.868720	1.125300	0.847410	0.011413	1.349800
27	0.407450	0.605530	1.423300	0.323490	1.393300
28	1.477600	1.196200	0.834050	1.465600	0.161960
29	0.269510	0.762190	0.665220	1.095000	0.994200
30	0.121540	0.004582	0.971640	0.476100	0.822970
31	0.065047	0.920190	0.223860	0.653380	0.148430
32	0.516280	0.075462	0.123790	0.414790	0.738230
33	1.412200	0.731970	0.486510	0.077169	0.400060
34	1.338100	0.434560	0.569770	1.133200	1.186100
35	0.292180	0.264880	1.341500	0.713830	0.622110
36	0.421000	0.814350	0.076858	0.143540	0.793510
37	1.468700	1.001700	0.954640	1.180100	0.848000
38	0.969310	0.113760	0.536980	0.807060	1.411400
39	0.813420	0.353800	1.470200	1.435800	1.077500
40	0.951620	0.278170	0.331140	1.332800	0.265550
41	1.409400	0.659160	1.194600	0.276410	1.281200
42	1.176900	0.219130	0.461250	0.950480	0.484260
43	0.040073	1.399000	0.640110	0.226400	1.095000
44	0.464430	0.803670	0.195950	1.013500	0.304760
45	0.690130	0.694200	0.692600	0.092788	0.957350
46	1.013800	0.172050	1.083200	1.492200	0.002612
47	1.256200	0.326790	0.048641	0.260750	0.433230
48	0.166260	0.551850	0.908860	1.390900	0.055920
49	1.215500	0.476710	0.747650	0.197950	0.075353
50	1.285700	0.535410	1.452400	0.444090	0.465490

Table S2: Scaling factors for each anthropogenic emission sector of the testing runs. Applied to all species within the sector. Sectors are residential (RES), industry (IND), land transport (TRA), agriculture (AGR), and power generation (ENE).

Run	RES	IND	TRA	AGR	ENE
**1**	0.800999	0.119784	1.370262	0.752336	0.336031
**2**	0.017744	0.523419	0.600339	0.476919	1.439264
**3**	1.005595	1.3623	1.147695	1.144138	1.135136
**4**	1.489352	0.889862	0.530681	0.001446	0.657881
**5**	0.340401	1.107743	0.265786	1.457797	0.181091

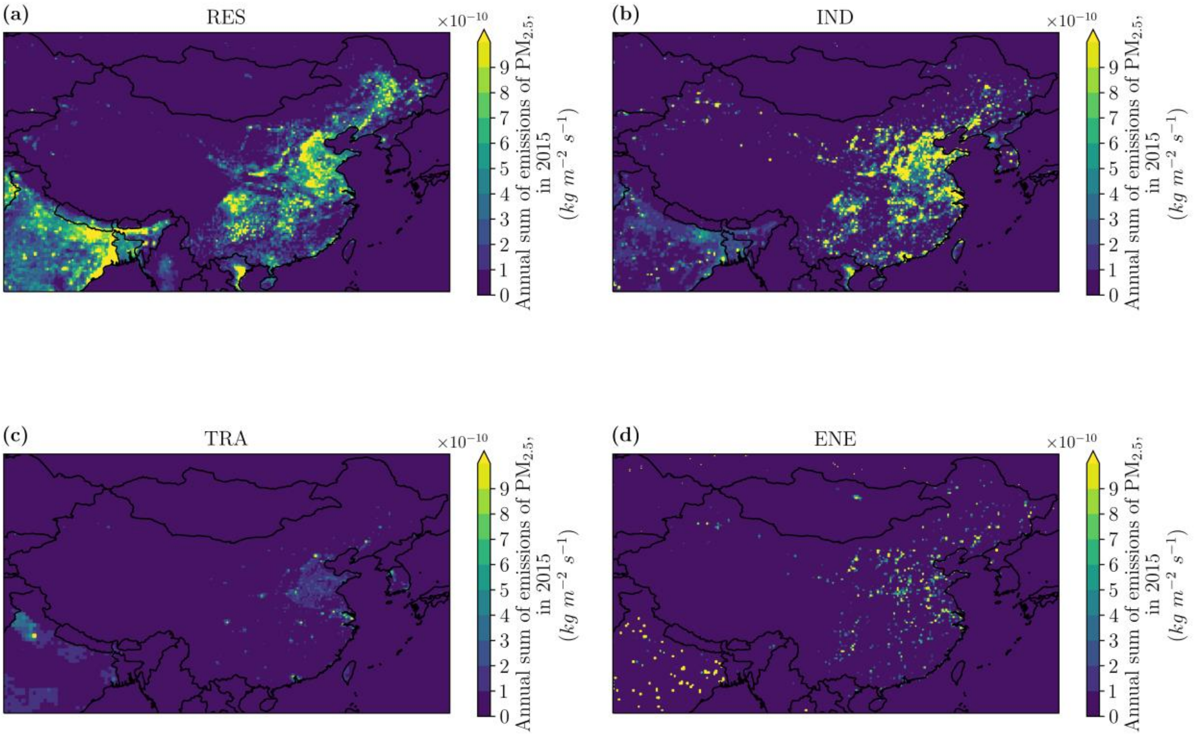

Figure S1: Anthropogenic emissions of fine particulate matter ($PM_{2.5}$) in 2015 across China from the (a) residential (RES), (b), industrial (IND), (c) land transport (TRA), and (d) power generation (ENE) sectors.

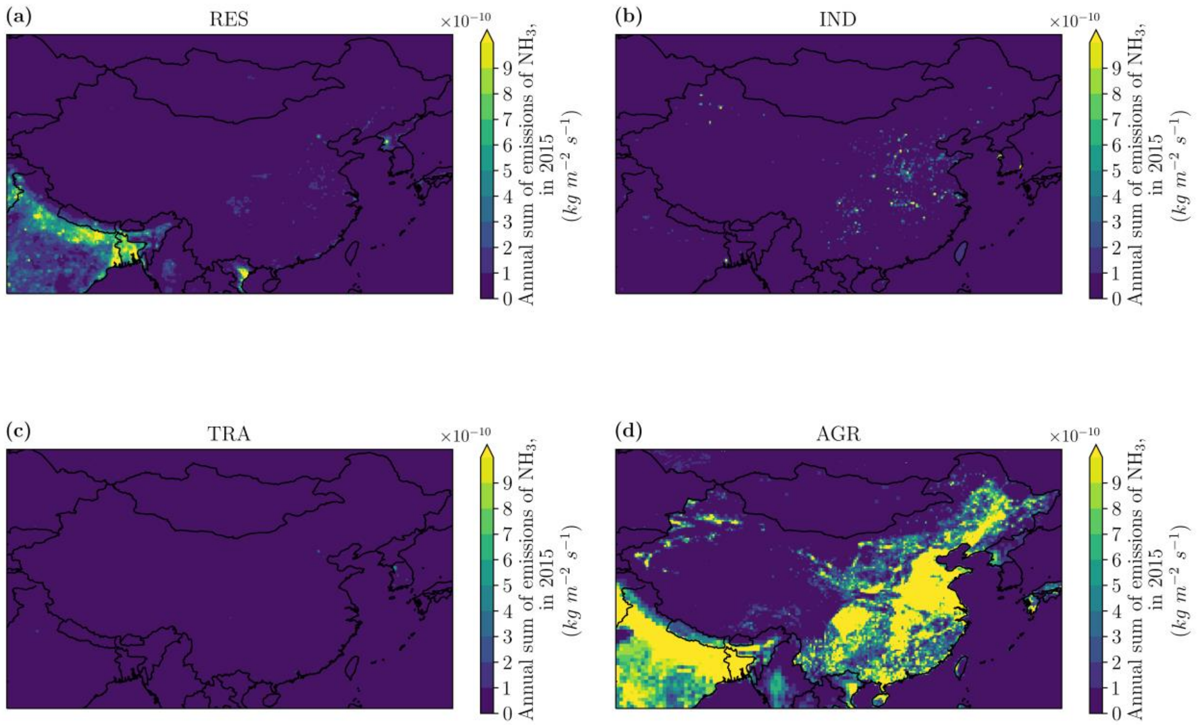

Figure S2: Anthropogenic emissions of ammonia (NH_3) in 2015 across China from the (a) residential (RES), (b), industrial (IND), (c) land transport (TRA), and (d) agricultural (AGR) sectors.

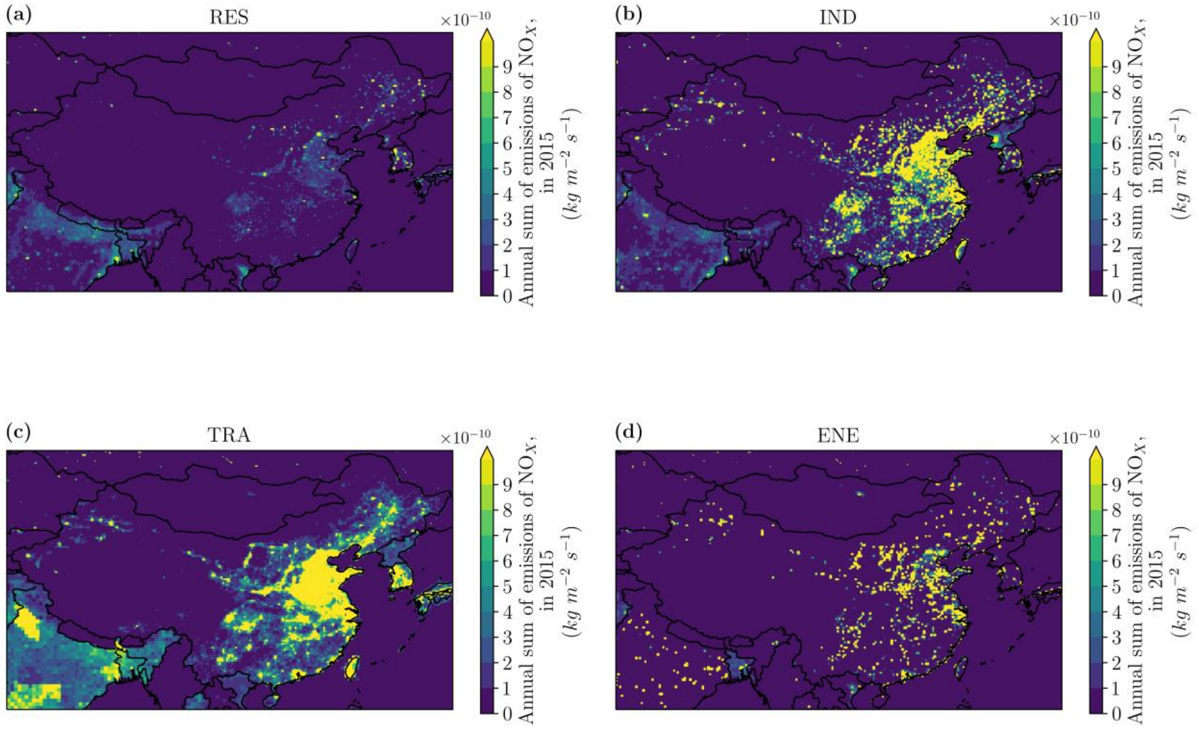

Figure S3: Anthropogenic emissions of nitrogen oxides (NO_X) in 2015 across China from the (a) residential (RES), (b), industrial (IND), (c) land transport (TRA), and (d) power generation (ENE) sectors.

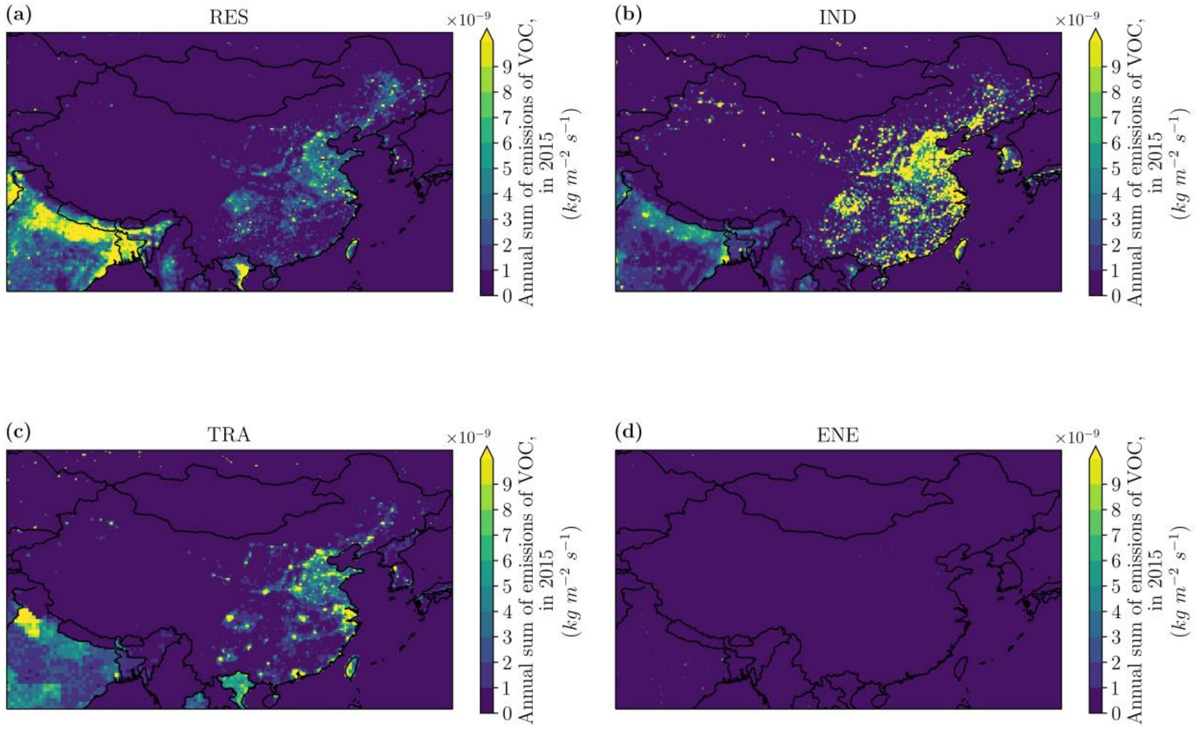

Figure S4: Anthropogenic emissions of non−methane volatile organic compounds (VOC) in 2015 across China from the (a) residential (RES), (b), industrial (IND), (c) land transport (TRA), and (d) power generation (ENE) sectors.

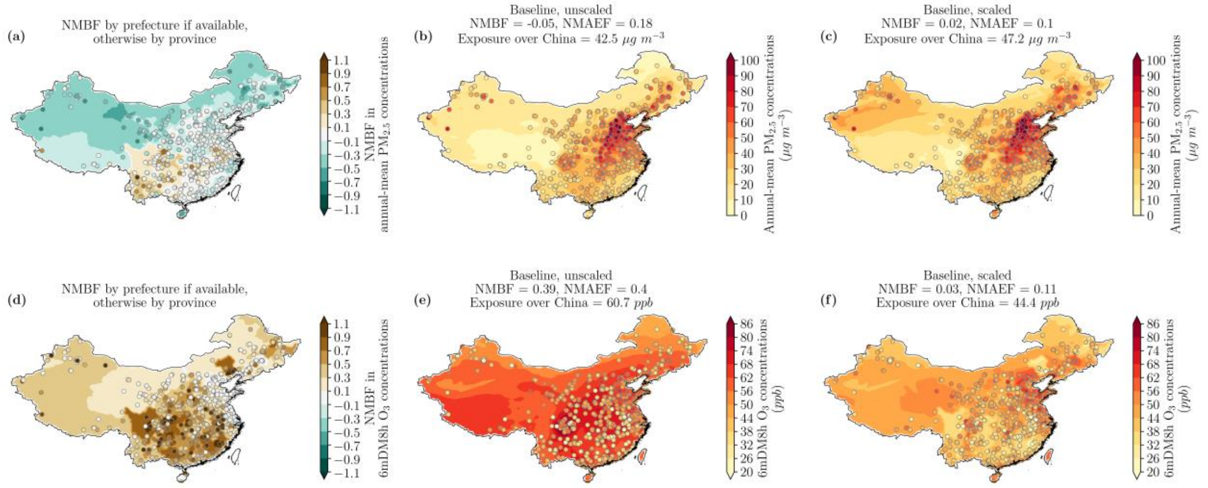

Figure S5: Evaluation of unscaled and scaled baseline simulator air quality concentrations. Regional evaluation metrics are normalised mean bias factor (NMBF) grouped by prefecture if available, otherwise by province for (a) for fine particulate matter ($PM_{2.5}$, annual−mean) and (d) ozone (O_3, maximum 6−monthly−mean daily−maximum 8−hour, 6mDM8h). Unscaled baseline concentrations for (b) $PM_{2.5}$ (NMBF = −0.05, normalised absolute error factor, NMAEF, = 0.18) and (e) O_3 (NMBF = 0.39, NMAEF = 0.40). Scaled baseline concentrations for (c) $PM_{2.5}$ (NMBF = 0.02, NMAEF, = 0.10) and (f) O_3 (NMBF = 0.03, NMAEF = 0.11).

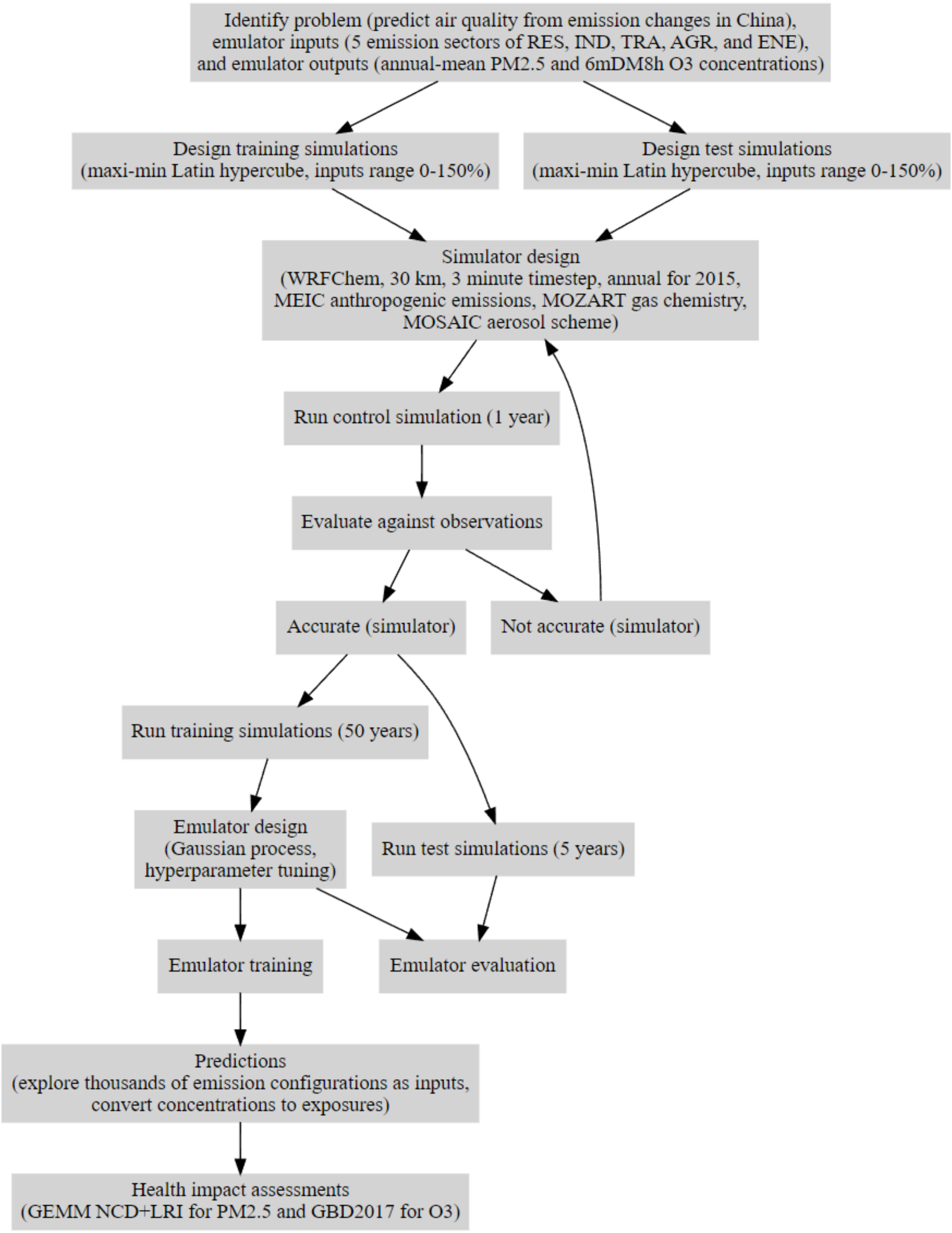

Figure S6: Directed acyclic graph of the emulator workflow. Information in brackets represents information that is specific to this work. The emulator inputs were anthropogenic emissions from the residential (RES), industrial (IND), land transport (TRA), agricultural (AGR), and power generation (ENE) sectors. The emulator outputs were annual–mean fine particulate matter ($PM_{2.5}$) concentrations and maximum 6–monthly–mean daily–maximum 8–hour (6mDM8h) ozone (O_3) concentrations. The simulator was WRFChem (Weather Research and Forecasting model online–coupled with Chemistry), with anthropogenic emissions from MEIC (Multi–resolution Emission Inventory for China), gas phase chemistry from MOZART (Model for Ozone and Related Chemical Tracers), and aerosol chemistry and physics from MOSAIC (Model for Simulating Aerosol Interactions and Chemistry). The health impact assessment used exposure–outcome associations from the GEMM (Global Exposure Mortality

Model) for non−accidental mortality (non−communicable disease, NCD, plus lower respiratory infections, LRI) for $PM_{2.5}$ exposure and from the Global Burden of Diseases, Injuries, and Risk Factors Study (GBD) from 2017 for O_3 exposure. See the Methods for more information on each step.

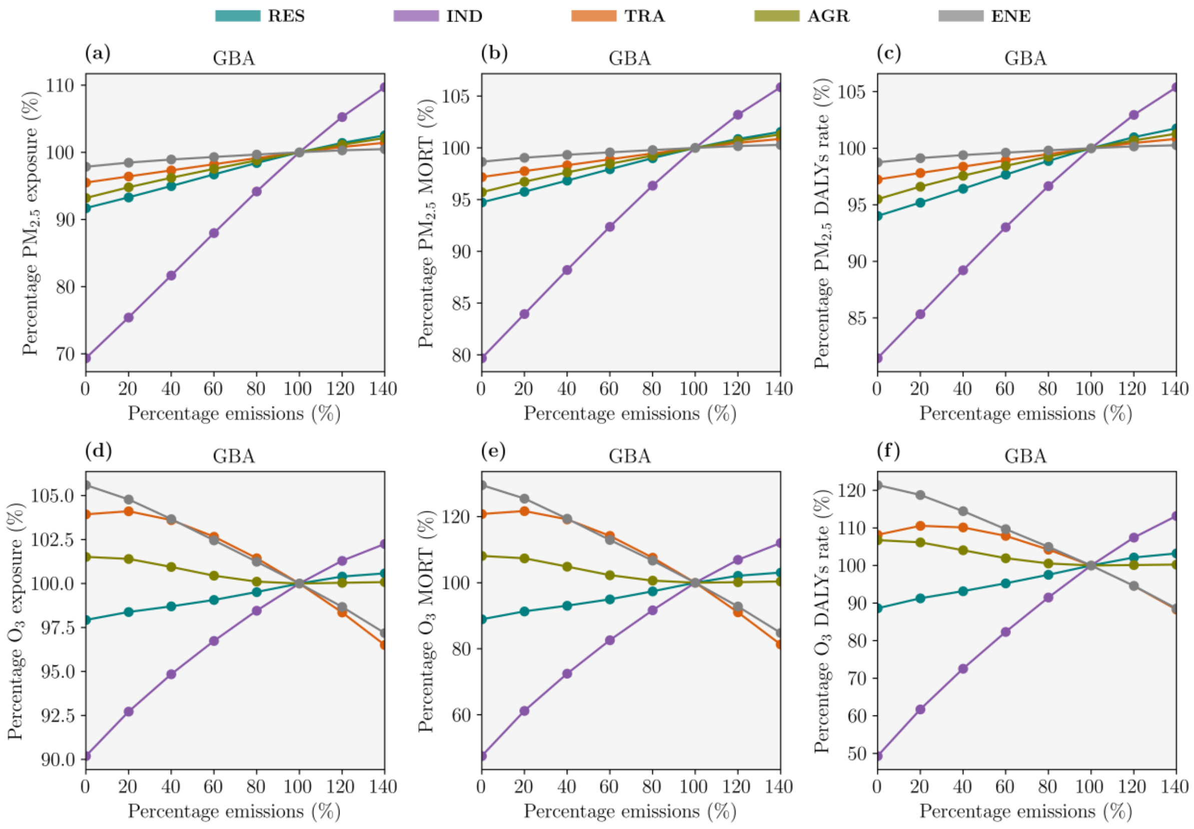

Figure S7: The fractional impact of individual emission changes in the Guangdong−Hong Kong−Macau Greater Bay Area (GBA) on (a) fine particulate matter ($PM_{2.5}$, annual−mean) exposure, (b) annual premature mortalities (MORT) from $PM_{2.5}$ exposure, (c) annual rate of disability−adjusted life years (DALYs) per 100,000 people from $PM_{2.5}$ exposure, (d) ozone (O_3, maximum 6−monthly−mean daily−maximum 8−hour, 6mDM8h) exposure, (e) annual MORT from O_3 exposure, and (f) annual rate of DALYs per 100,000 people from O_3 exposure. The five emission sectors are residential (RES), industry (IND), land transport (TRA), agriculture (AGR), and power generation (ENE).

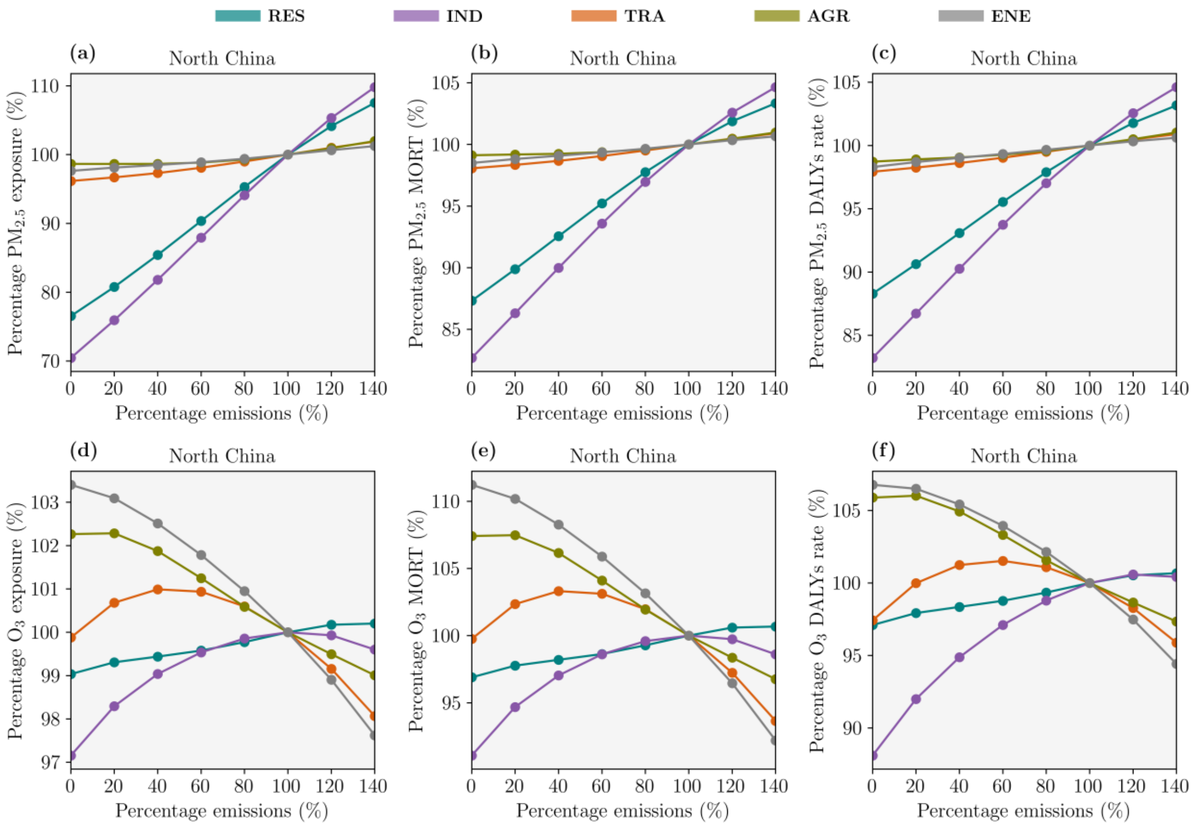

Figure S8: The fractional impact of individual emission changes in North China on (a) fine particulate matter ($PM_{2.5}$, annual−mean) exposure, (b) annual premature mortalities (MORT) from $PM_{2.5}$ exposure, (c) annual rate of disability−adjusted life years (DALYs) per 100,000 people from $PM_{2.5}$ exposure, (d) ozone (O_3, maximum 6−monthly−mean daily−maximum 8−hour, 6mDM8h) exposure, (e) annual MORT from O_3 exposure, and (f) annual rate of DALYs per 100,000 people from O_3 exposure. The five emission sectors are residential (RES), industry (IND), land transport (TRA), agriculture (AGR), and power generation (ENE).

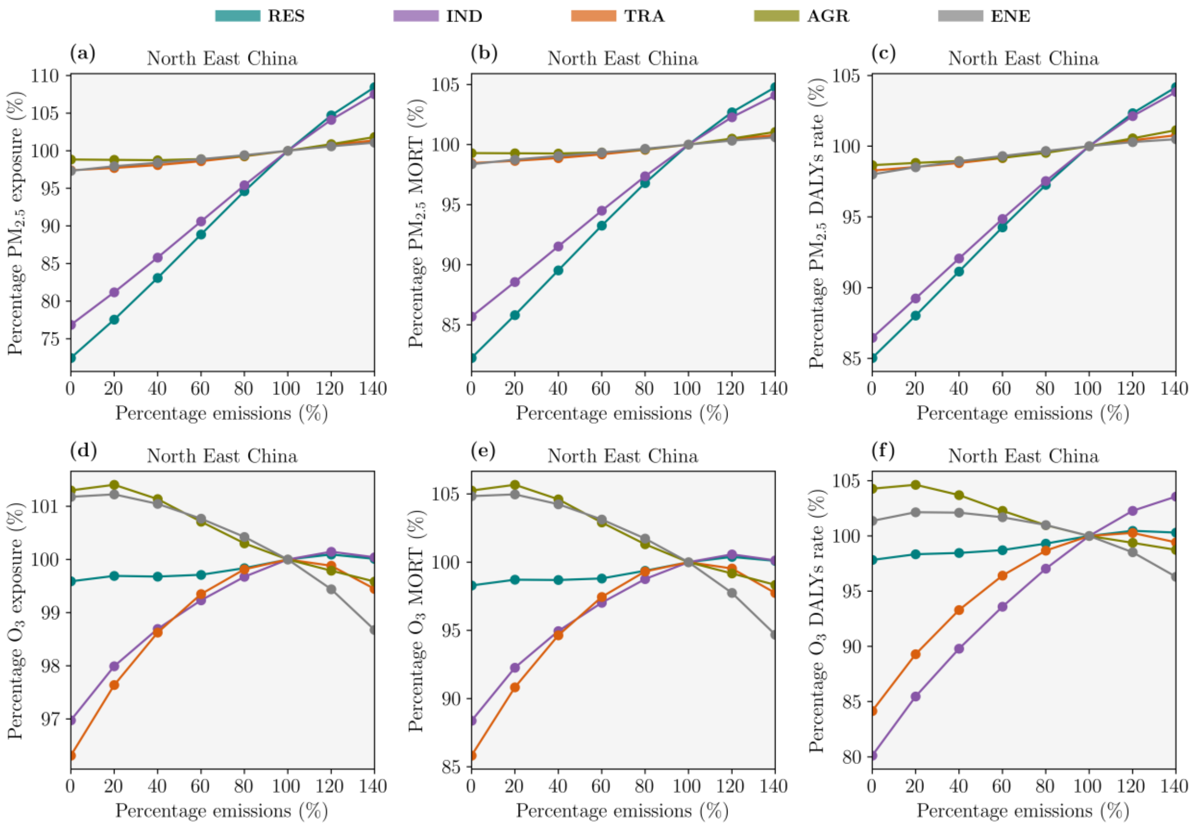

Figure S9: The fractional impact of individual emission changes in North East China on (a) fine particulate matter ($PM_{2.5}$, annual–mean) exposure, (b) annual premature mortalities (MORT) from $PM_{2.5}$ exposure, (c) annual rate of disability–adjusted life years (DALYs) per 100,000 people from $PM_{2.5}$ exposure, (d) ozone (O_3, maximum 6–monthly–mean daily–maximum 8–hour, 6mDM8h) exposure, (e) annual MORT from O_3 exposure, and (f) annual rate of DALYs per 100,000 people from O_3 exposure. The five emission sectors are residential (RES), industry (IND), land transport (TRA), agriculture (AGR), and power generation (ENE).

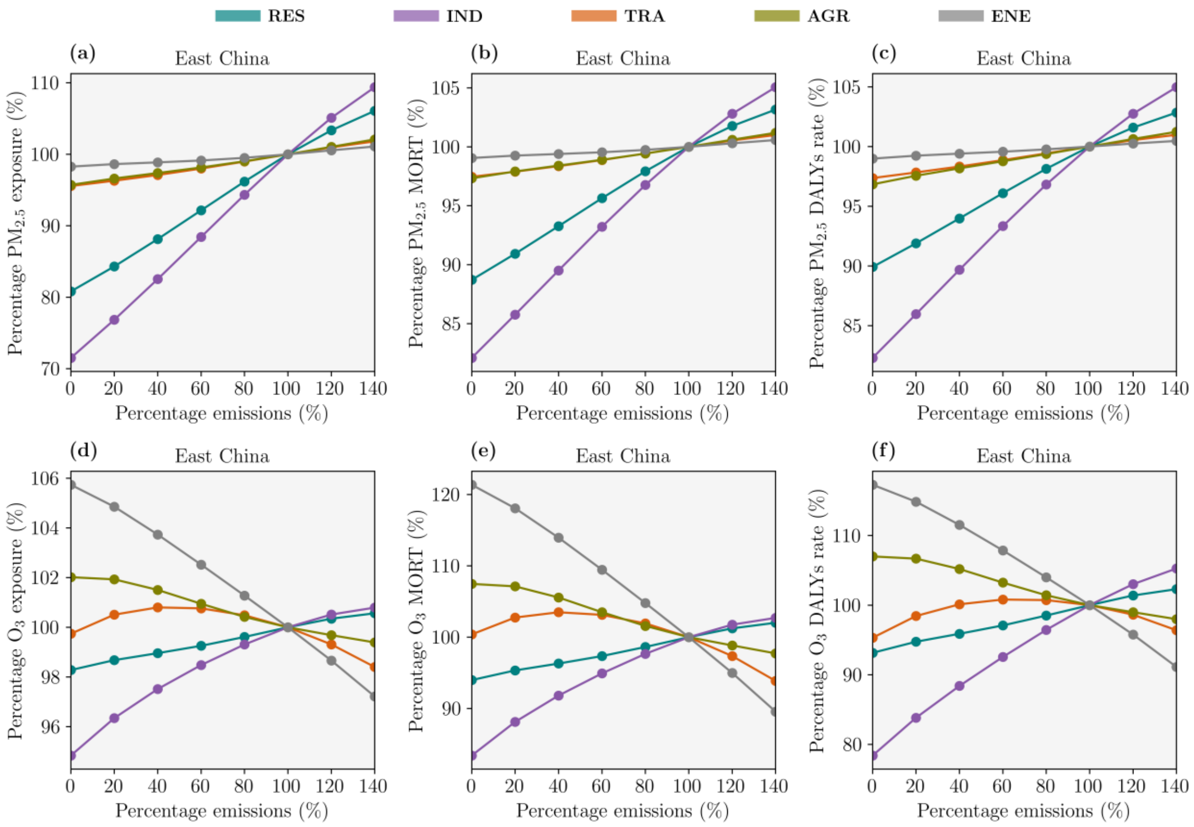

Figure S10: The fractional impact of individual emission changes in East China on (a) fine particulate matter ($PM_{2.5}$, annual−mean) exposure, (b) annual premature mortalities (MORT) from $PM_{2.5}$ exposure, (c) annual rate of disability−adjusted life years (DALYs) per 100,000 people from $PM_{2.5}$ exposure, (d) ozone (O_3, maximum 6−monthly−mean daily−maximum 8−hour, 6mDM8h) exposure, (e) annual MORT from O_3 exposure, and (f) annual rate of DALYs per 100,000 people from O_3 exposure. The five emission sectors are residential (RES), industry (IND), land transport (TRA), agriculture (AGR), and power generation (ENE).

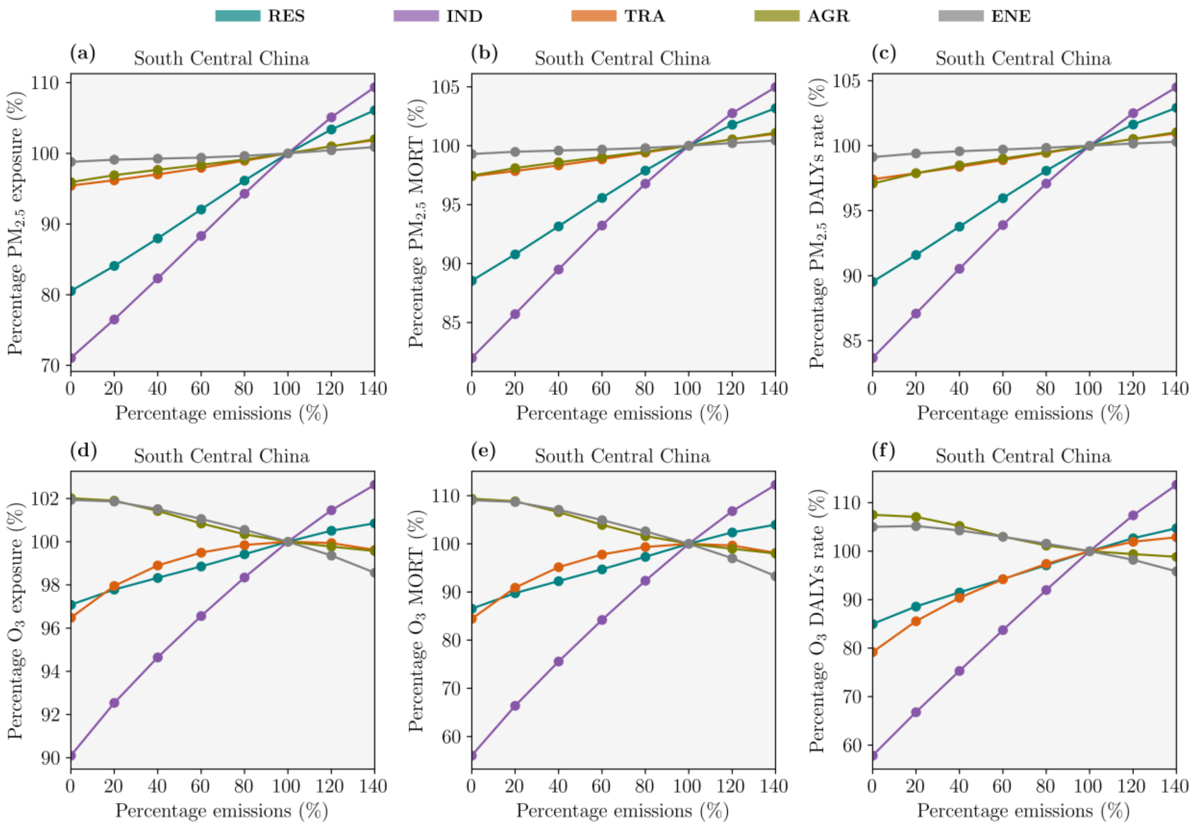

Figure S11: The fractional impact of individual emission changes in South Central China on (a) fine particulate matter ($PM_{2.5}$, annual−mean) exposure, (b) annual premature mortalities (MORT) from $PM_{2.5}$ exposure, (c) annual rate of disability−adjusted life years (DALYs) per 100,000 people from $PM_{2.5}$ exposure, (d) ozone (O_3, maximum 6−monthly−mean daily−maximum 8−hour, 6mDM8h) exposure, (e) annual MORT from O_3 exposure, and (f) annual rate of DALYs per 100,000 people from O_3 exposure. The five emission sectors are residential (RES), industry (IND), land transport (TRA), agriculture (AGR), and power generation (ENE).

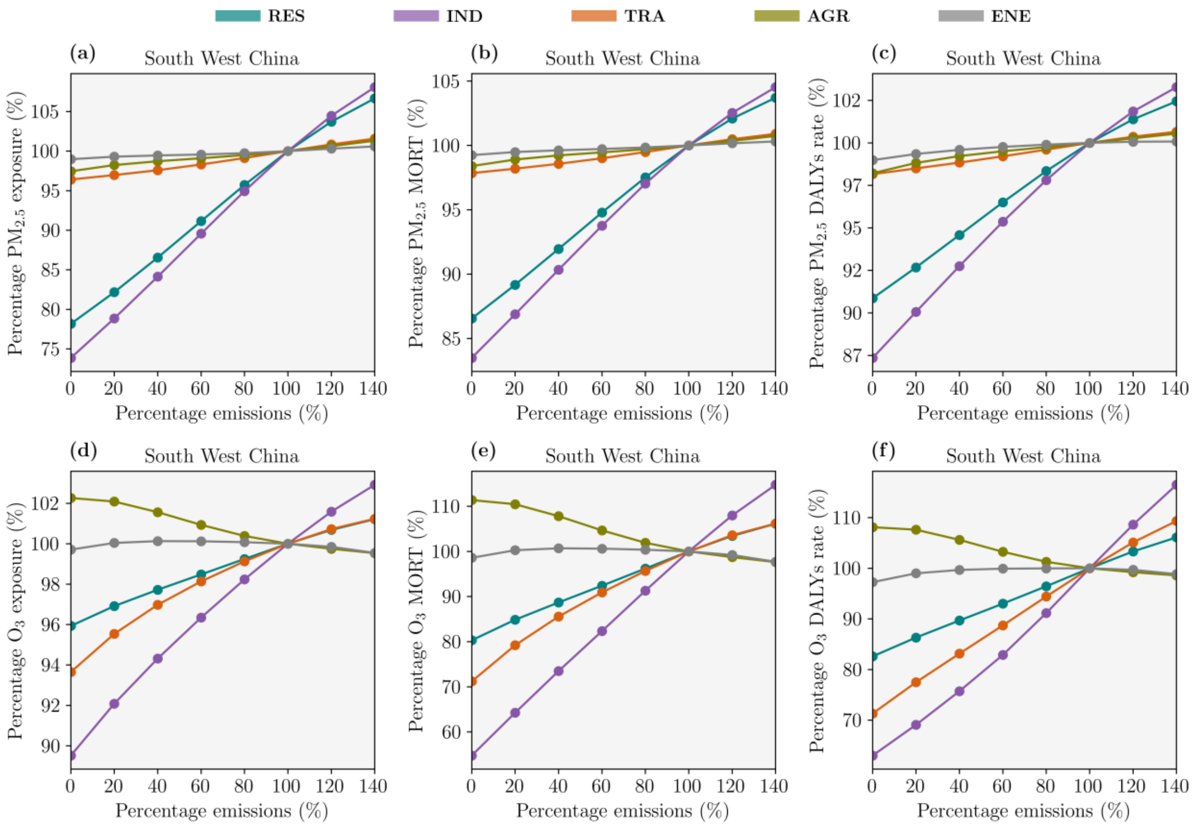

Figure S12: The fractional impact of individual emission changes in South West China on (a) fine particulate matter ($PM_{2.5}$, annual−mean) exposure, (b) annual premature mortalities (MORT) from $PM_{2.5}$ exposure, (c) annual rate of disability−adjusted life years (DALYs) per 100,000 people from $PM_{2.5}$ exposure, (d) ozone (O_3, maximum 6−monthly−mean daily−maximum 8−hour, 6mDM8h) exposure, (e) annual MORT from O_3 exposure, and (f) annual rate of DALYs per 100,000 people from O_3 exposure. The five emission sectors are residential (RES), industry (IND), land transport (TRA), agriculture (AGR), and power generation (ENE).

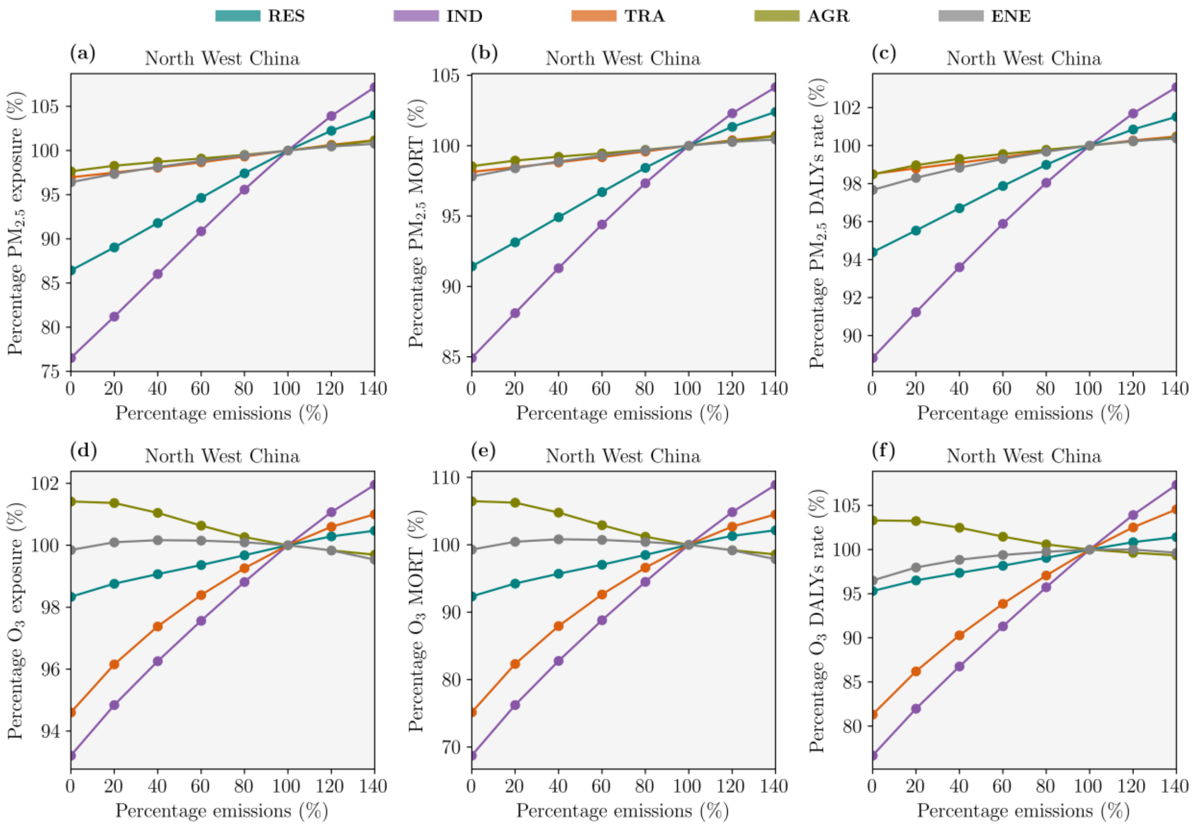

Figure S13: The fractional impact of individual emission changes in North West China on (a) fine particulate matter ($PM_{2.5}$, annual−mean) exposure, (b) annual premature mortalities (MORT) from $PM_{2.5}$ exposure, (c) annual rate of disability−adjusted life years (DALYs) per 100,000 people from $PM_{2.5}$ exposure, (d) ozone (O_3, maximum 6−monthly−mean daily−maximum 8−hour, 6mDM8h) exposure, (e) annual MORT from O_3 exposure, and (f) annual rate of DALYs per 100,000 people from O_3 exposure. The five emission sectors are residential (RES), industry (IND), land transport (TRA), agriculture (AGR), and power generation (ENE).

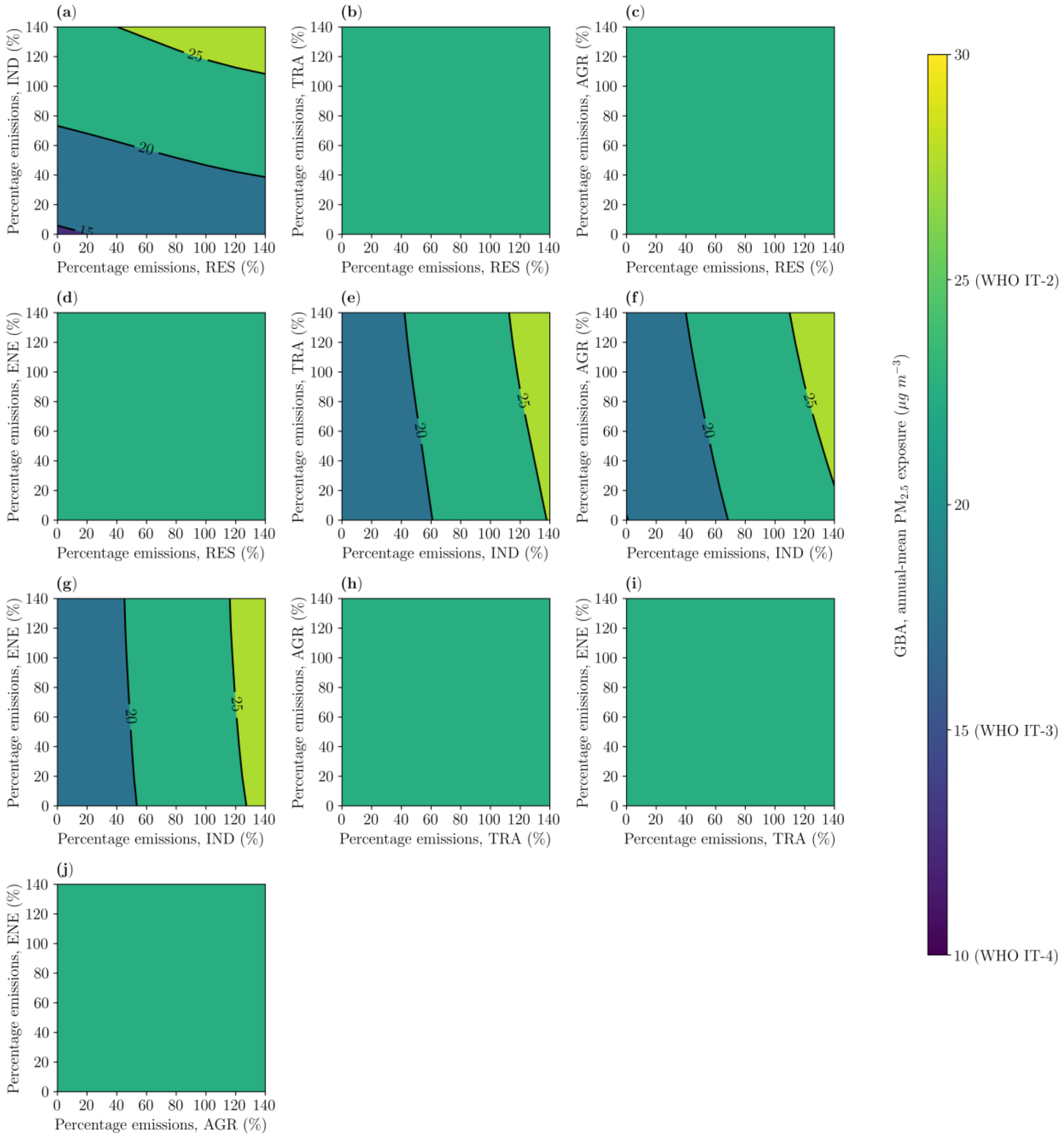

Figure S14: The impact of variations in two emission sectors on fine particulate matter ($PM_{2.5}$, annual−mean) exposure for Guangdong−Hong Kong−Macau Greater Bay Area (GBA) from (a) residential (RES) and industry (IND), (b) RES and land transport (TRA), (c) RES and agriculture (AGR), (d) RES and power generation (ENE), (e) IND and TRA, (f) IND and AGR, (g) IND and ENE, (h) TRA and AGR, (i) TRA and ENE, and (j) AGR and ENE emissions. Air quality targets shown for the World Health Organization's (WHO) Air Quality Guideline (AQG, 5 μg m^{-3}), Interim Target 1 (IT−1, 35 μg m^{-3}), Interim Target 2 (IT−2, 25 μg m^{-3}), Interim Target 3 (IT−3, 15 μg m^{-3}), Interim Target 4 (IT−4, 10 μg m^{-3}), and China's National Air Quality Target (NAQT, 35 μg m^{-3}).

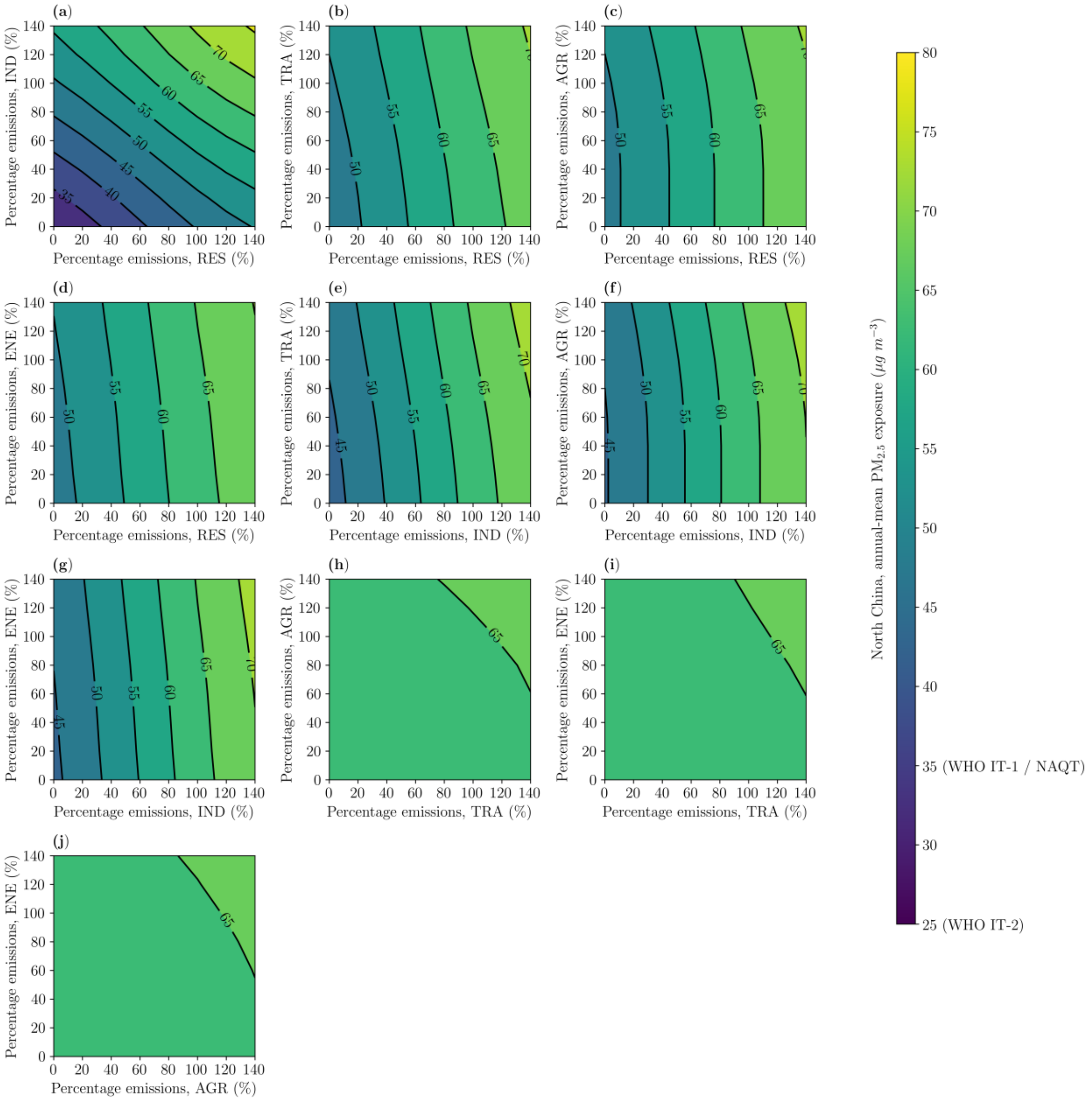

Figure S15: The impact of variations in two emission sectors on fine particulate matter ($PM_{2.5}$, annual−mean) exposure for North China from (a) residential (RES) and industry (IND), (b) RES and land transport (TRA), (c) RES and agriculture (AGR), (d) RES and power generation (ENE), (e) IND and TRA, (f) IND and AGR, (g) IND and ENE, (h) TRA and AGR, (i) TRA and ENE, and (j) AGR and ENE emissions. Air quality targets shown for the World Health Organization's (WHO) Air Quality Guideline (AQG, 5 μg m^{-3}), Interim Target 1 (IT−1, 35 μg m^{-3}), Interim Target 2 (IT−2, 25 μg m^{-3}), Interim Target 3 (IT−3, 15 μg m^{-3}), Interim Target 4 (IT−4, 10 μg m^{-3}), and China's National Air Quality Target (NAQT, 35 μg m^{-3}).

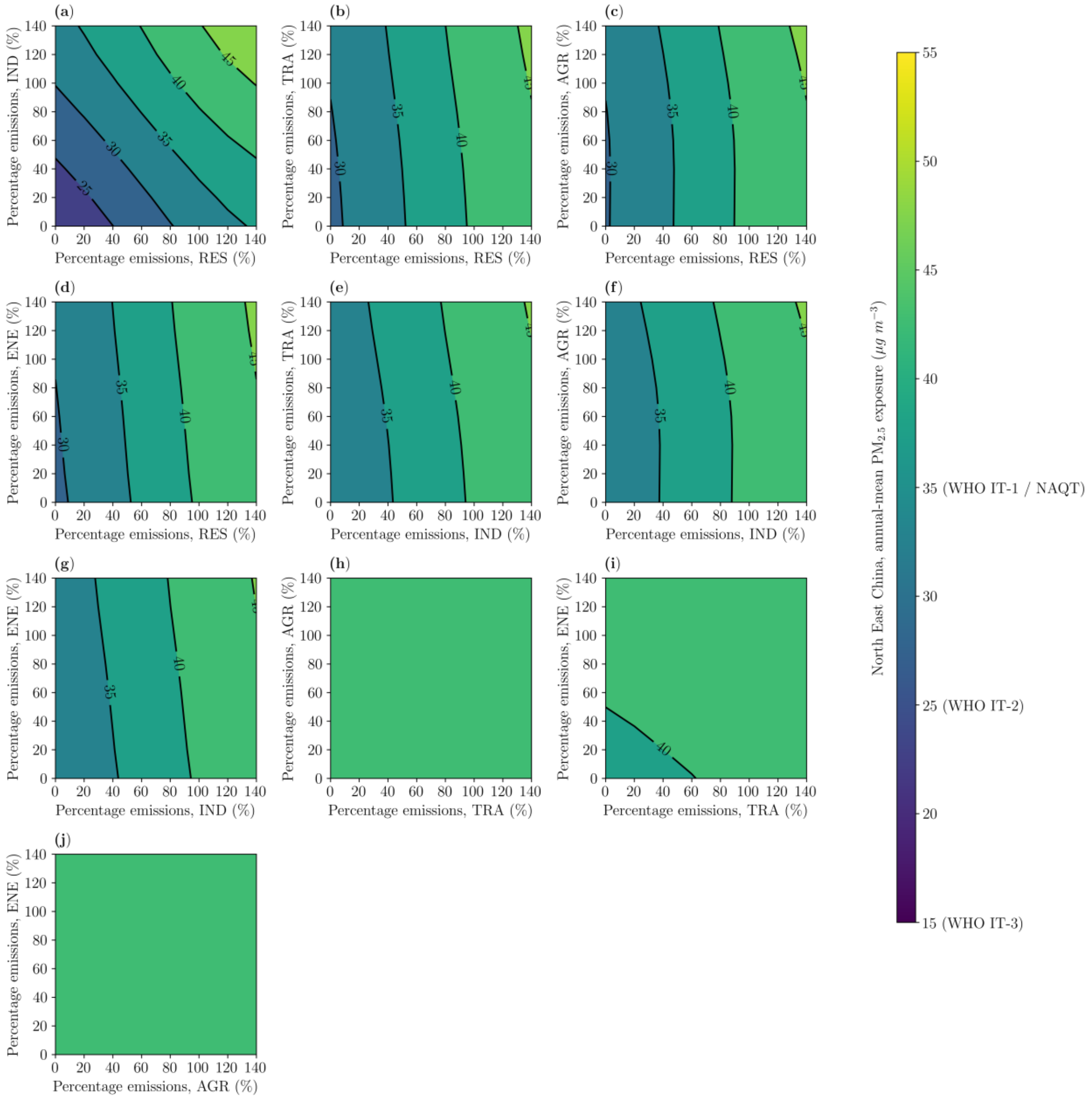

Figure S16: The impact of variations in two emission sectors on fine particulate matter ($PM_{2.5}$, annual−mean) exposure for North East China from (a) residential (RES) and industry (IND), (b) RES and land transport (TRA), (c) RES and agriculture (AGR), (d) RES and power generation (ENE), (e) IND and TRA, (f) IND and AGR, (g) IND and ENE, (h) TRA and AGR, (i) TRA and ENE, and (j) AGR and ENE emissions. Air quality targets shown for the World Health Organization's (WHO) Air Quality Guideline (AQG, 5 μg m^{-3}), Interim Target 1 (IT−1, 35 μg m^{-3}), Interim Target 2 (IT−2, 25 μg m^{-3}), Interim Target 3 (IT−3, 15 μg m^{-3}), Interim Target 4 (IT−4, 10 μg m^{-3}), and China's National Air Quality Target (NAQT, 35 μg m^{-3}).

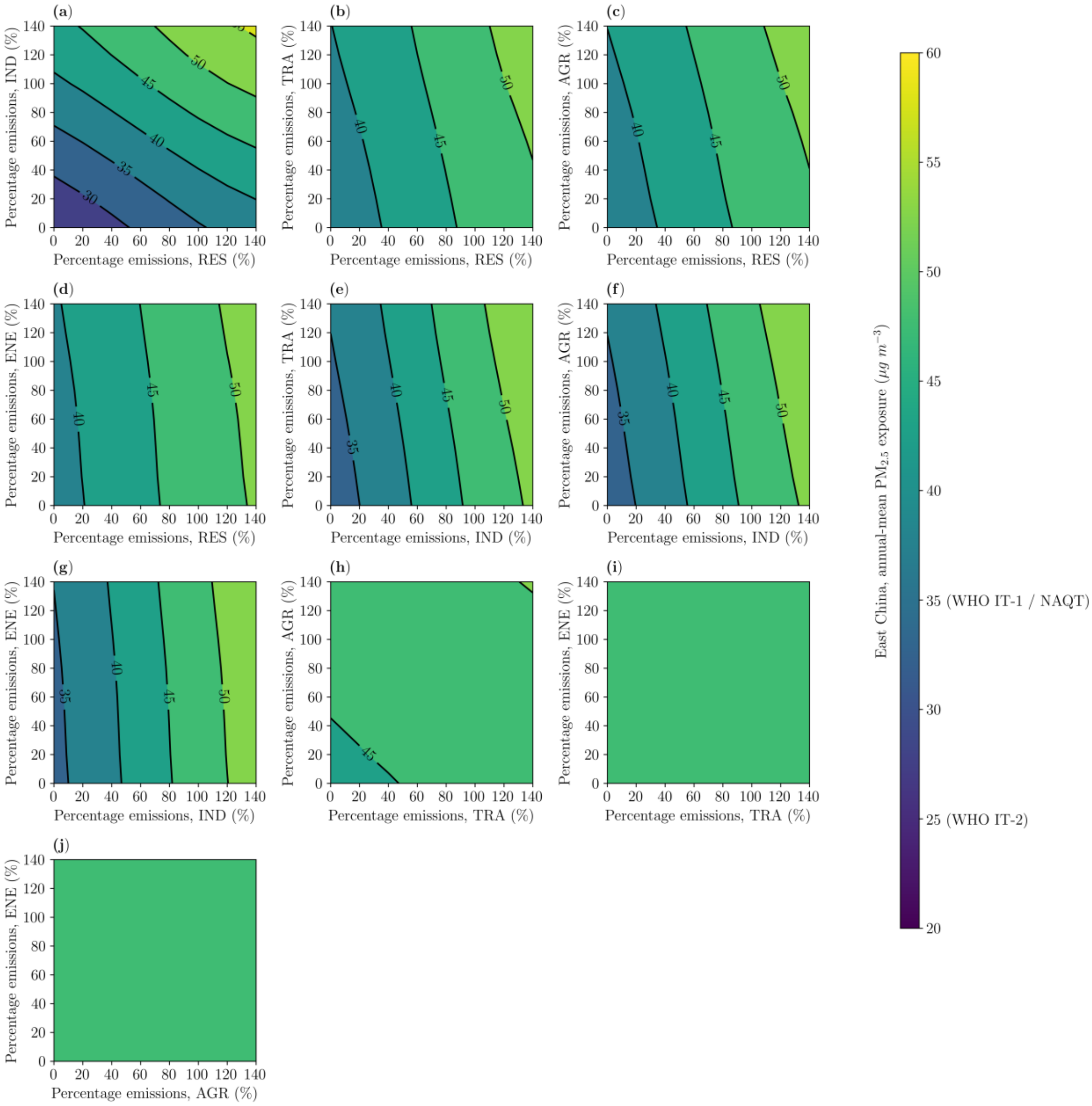

Figure S17: The impact of variations in two emission sectors on fine particulate matter ($PM_{2.5}$, annual−mean) exposure for East China from (a) residential (RES) and industry (IND), (b) RES and land transport (TRA), (c) RES and agriculture (AGR), (d) RES and power generation (ENE), (e) IND and TRA, (f) IND and AGR, (g) IND and ENE, (h) TRA and AGR, (i) TRA and ENE, and (j) AGR and ENE emissions. Air quality targets shown for the World Health Organization's (WHO) Air Quality Guideline (AQG, 5 µg m^{-3}), Interim Target 1 (IT−1, 35 µg m^{-3}), Interim Target 2 (IT−2, 25 µg m^{-3}), Interim Target 3 (IT−3, 15 µg m^{-3}), Interim Target 4 (IT−4, 10 µg m^{-3}), and China's National Air Quality Target (NAQT, 35 µg m^{-3}).

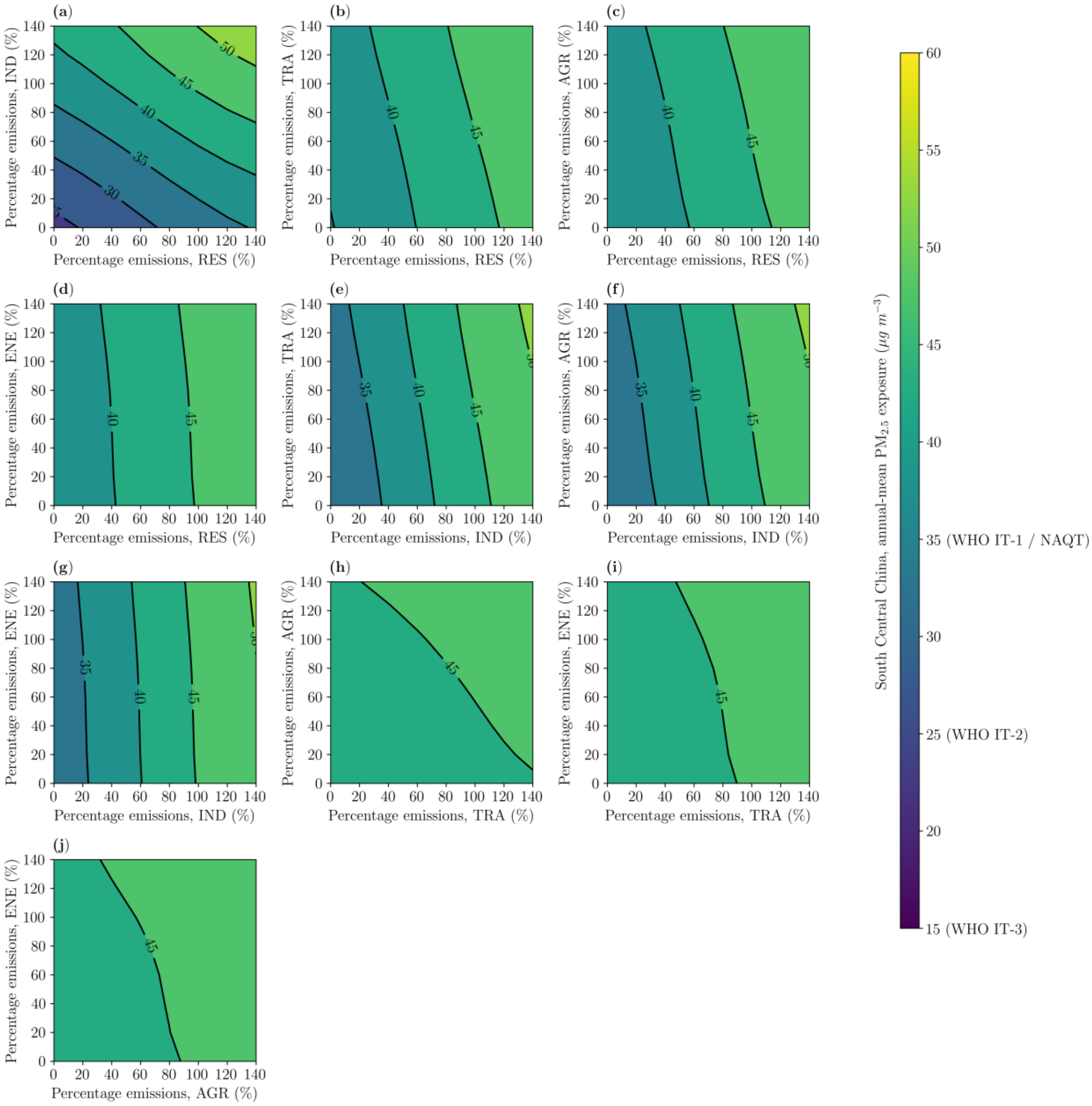

Figure S18: The impact of variations in two emission sectors on fine particulate matter ($PM_{2.5}$, annual−mean) exposure for South Central China from (a) residential (RES) and industry (IND), (b) RES and land transport (TRA), (c) RES and agriculture (AGR), (d) RES and power generation (ENE), (e) IND and TRA, (f) IND and AGR, (g) IND and ENE, (h) TRA and AGR, (i) TRA and ENE, and (j) AGR and ENE emissions. Air quality targets shown for the World Health Organization's (WHO) Air Quality Guideline (AQG, 5 μg m^{-3}), Interim Target 1 (IT−1, 35 μg m^{-3}), Interim Target 2 (IT−2, 25 μg m^{-3}), Interim Target 3 (IT−3, 15 μg m^{-3}), Interim Target 4 (IT−4, 10 μg m^{-3}), and China's National Air Quality Target (NAQT, 35 μg m^{-3}).

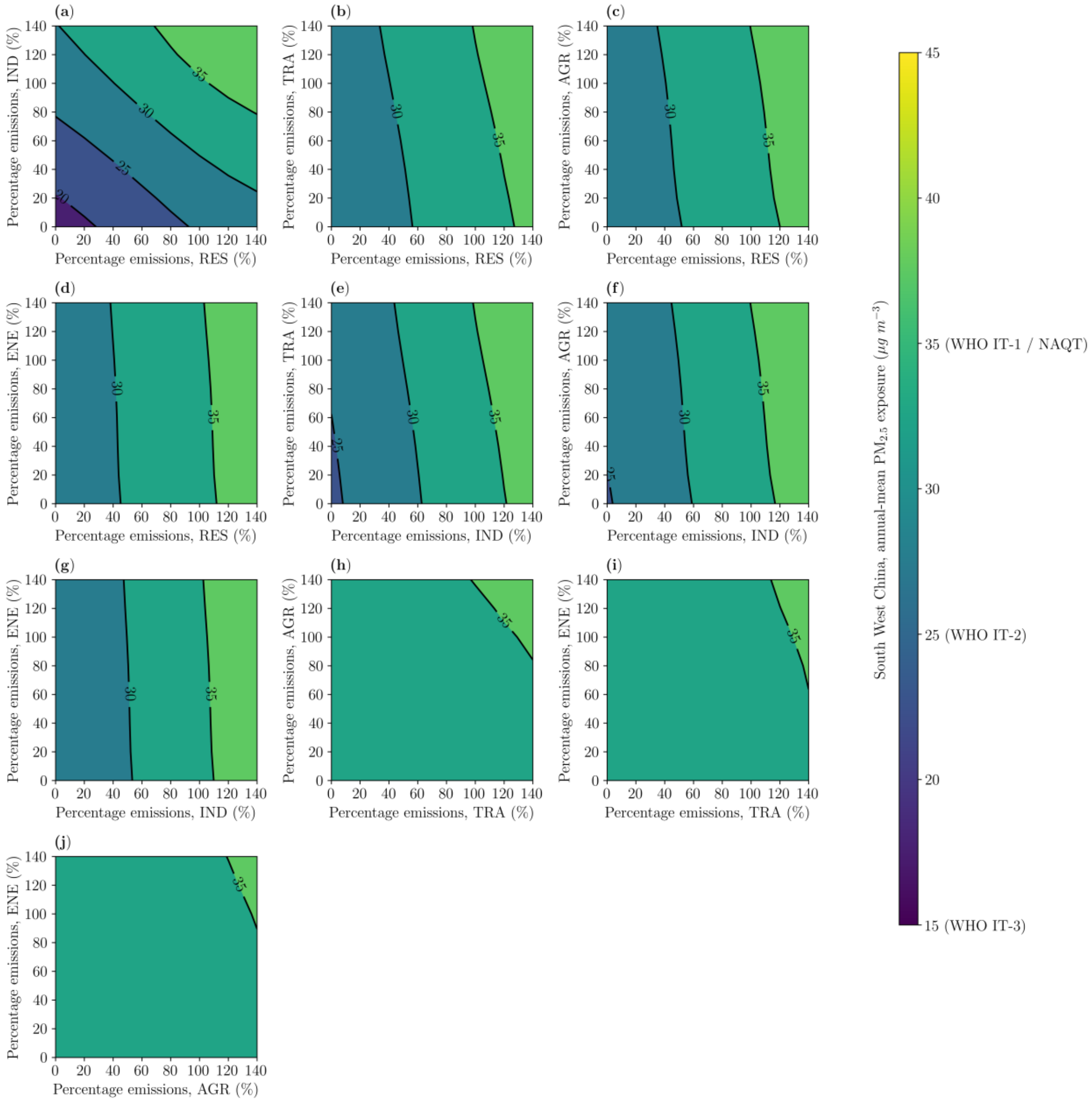

Figure S19: The impact of variations in two emission sectors on fine particulate matter ($PM_{2.5}$, annual−mean) exposure for South West China from (a) residential (RES) and industry (IND), (b) RES and land transport (TRA), (c) RES and agriculture (AGR), (d) RES and power generation (ENE), (e) IND and TRA, (f) IND and AGR, (g) IND and ENE, (h) TRA and AGR, (i) TRA and ENE, and (j) AGR and ENE emissions. Air quality targets shown for the World Health Organization's (WHO) Air Quality Guideline (AQG, 5 µg m^{-3}), Interim Target 1 (IT−1, 35 µg m^{-3}), Interim Target 2 (IT−2, 25 µg m^{-3}), Interim Target 3 (IT−3, 15 µg m^{-3}), Interim Target 4 (IT−4, 10 µg m^{-3}), and China's National Air Quality Target (NAQT, 35 µg m^{-3}).

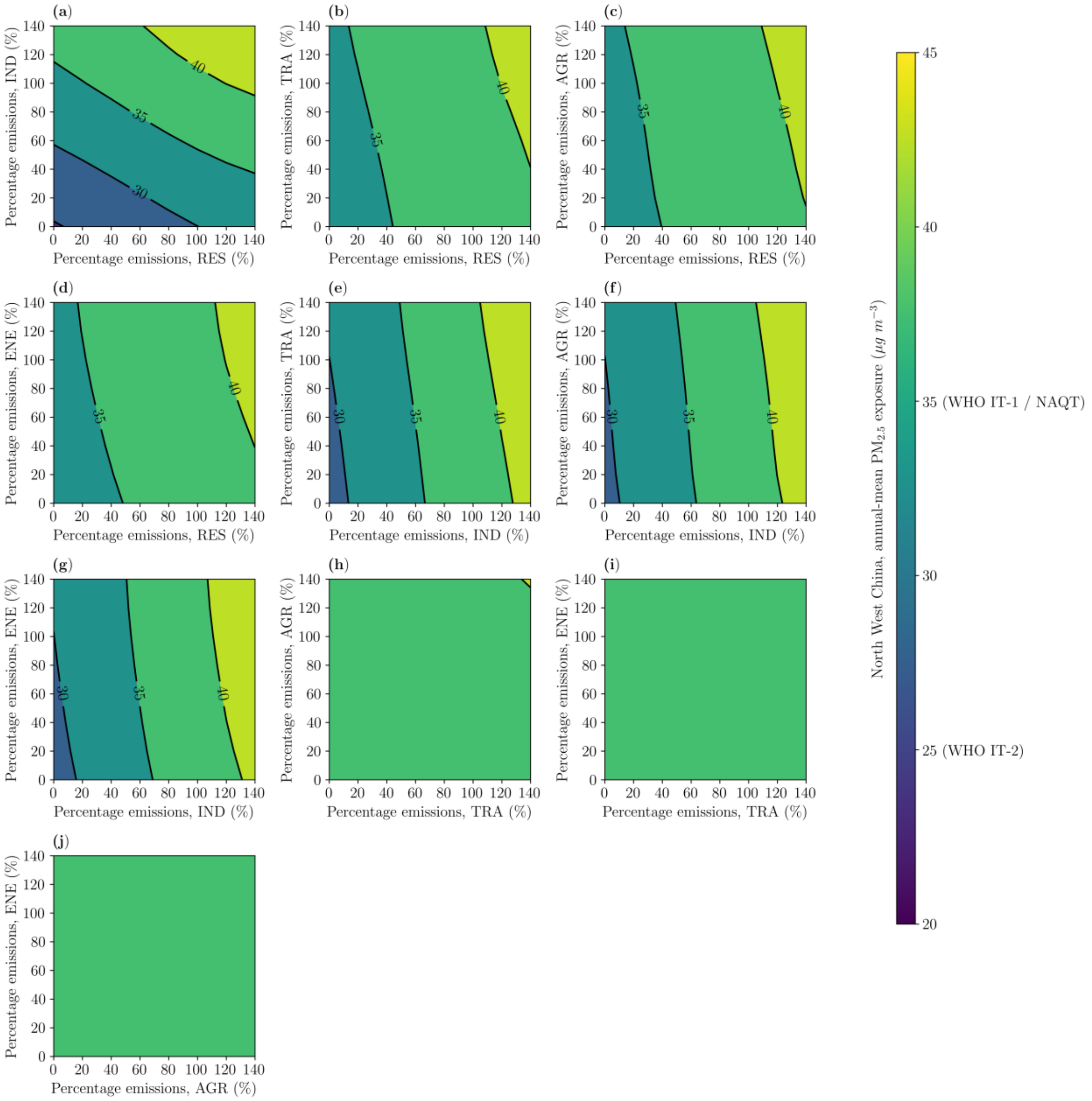

Figure S20: The impact of variations in two emission sectors on fine particulate matter ($PM_{2.5}$, annual−mean) exposure for North West China from (a) residential (RES) and industry (IND), (b) RES and land transport (TRA), (c) RES and agriculture (AGR), (d) RES and power generation (ENE), (e) IND and TRA, (f) IND and AGR, (g) IND and ENE, (h) TRA and AGR, (i) TRA and ENE, and (j) AGR and ENE emissions. Air quality targets shown for the World Health Organization's (WHO) Air Quality Guideline (AQG, 5 μg m^{-3}), Interim Target 1 (IT−1, 35 μg m^{-3}), Interim Target 2 (IT−2, 25 μg m^{-3}), Interim Target 3 (IT−3, 15 μg m^{-3}), Interim Target 4 (IT−4, 10 μg m^{-3}), and China's National Air Quality Target (NAQT, 35 μg m^{-3}).

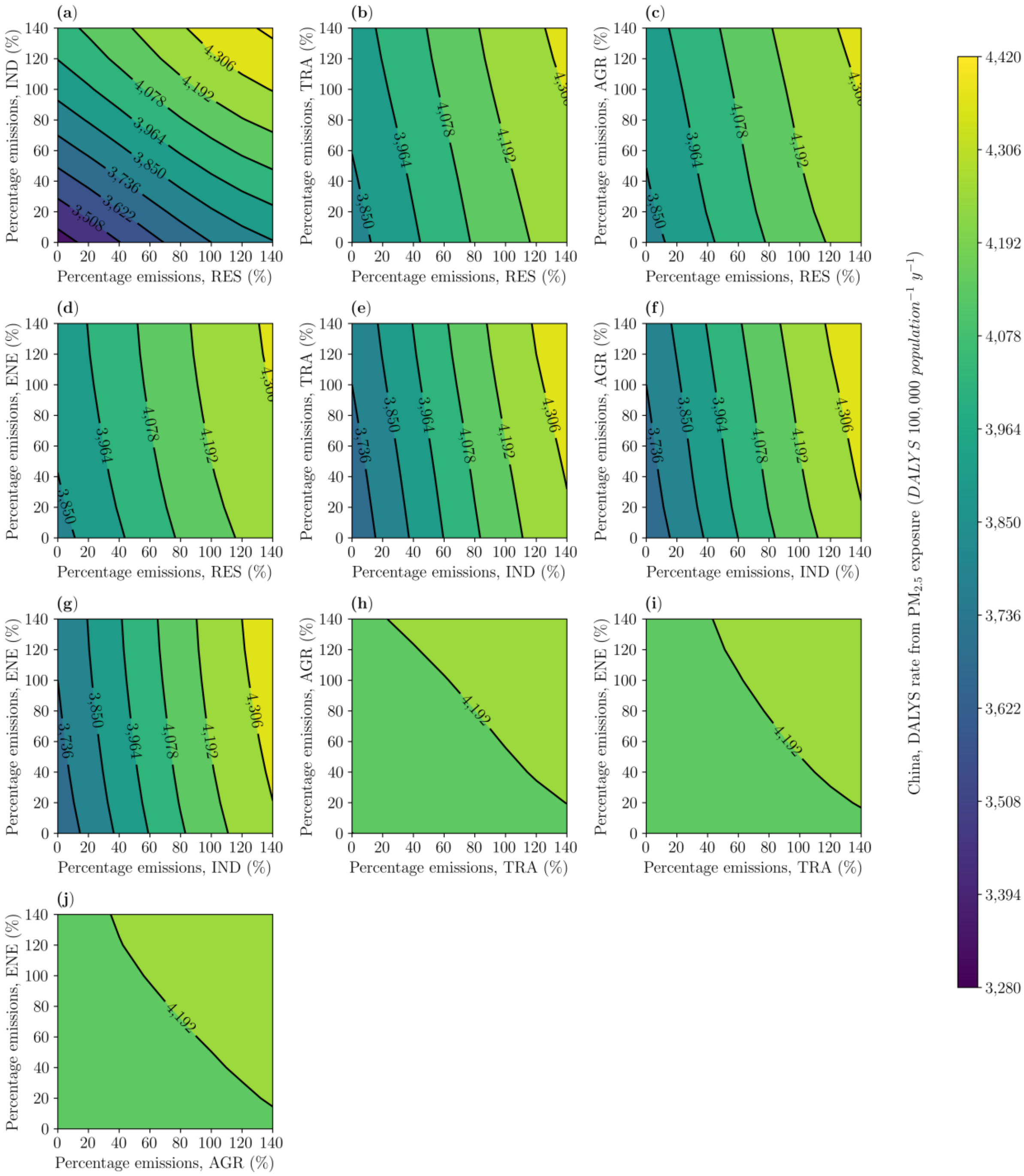

Figure S21: The impact of variations in two emission sectors on the disease burden (rate of disability−adjusted life years, DALYs, per 100,000 people per year) from fine particulate matter ($PM_{2.5}$, annual−mean) exposure for China from (a) residential (RES) and industry (IND), (b) RES and land transport (TRA), (c) RES and agriculture (AGR), (d) RES and power generation (ENE), (e) IND and TRA, (f) IND and AGR, (g) IND and ENE, (h) TRA and AGR, (i) TRA and ENE, and (j) AGR and ENE emissions.

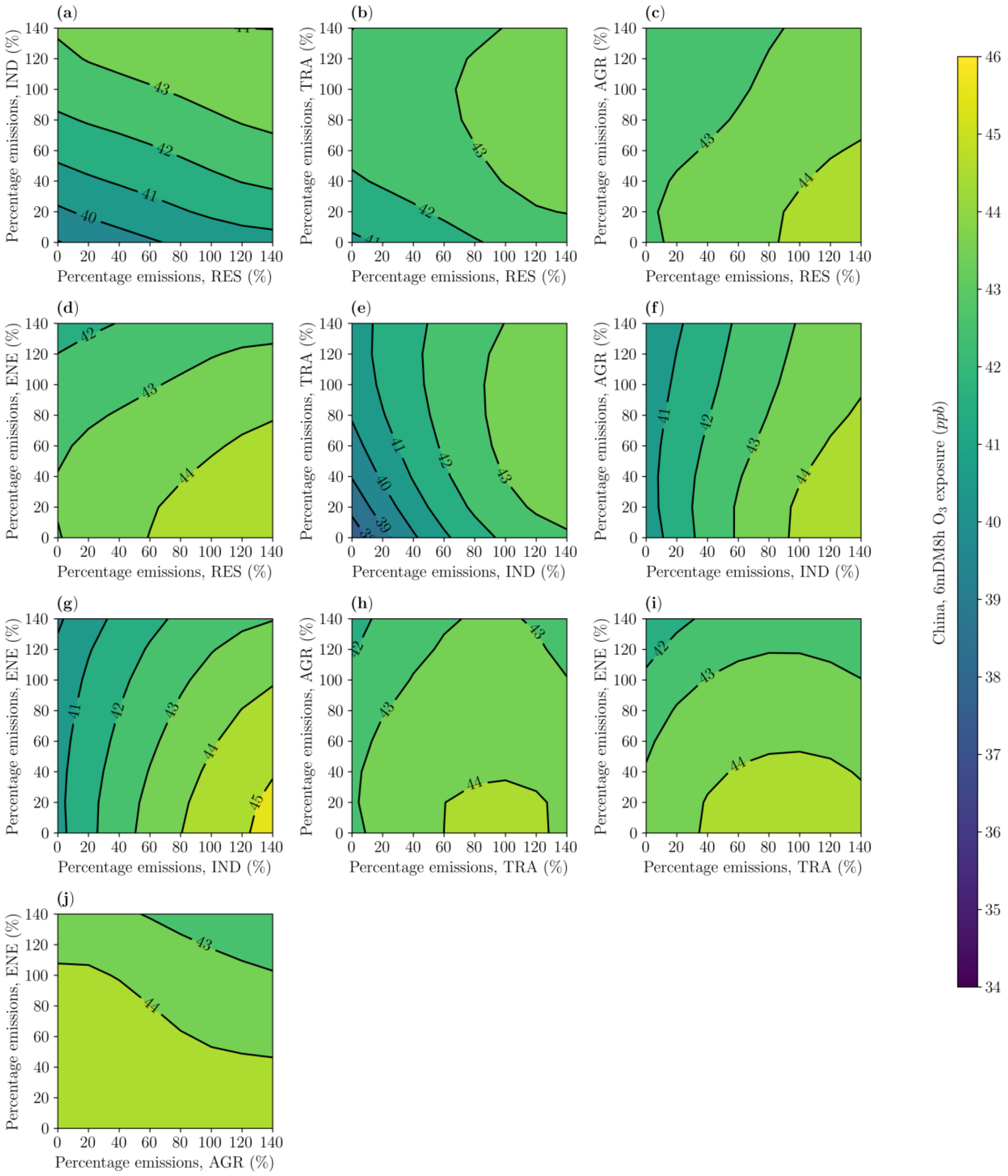

Figure S22: The impact of variations in two emission sectors on ozone (O_3, maximum 6−monthly−mean daily−maximum 8−hour, 6mDM8h) exposure in China for 2015 from (a) residential (RES) and industry (IND), (b) RES and land transport (TRA), (c) RES and agriculture (AGR), (d) RES and power generation (ENE), (e) IND and TRA, (f) IND and AGR, (g) IND and ENE, (h) TRA and AGR, (i) TRA and ENE, and (j) AGR and ENE emissions.

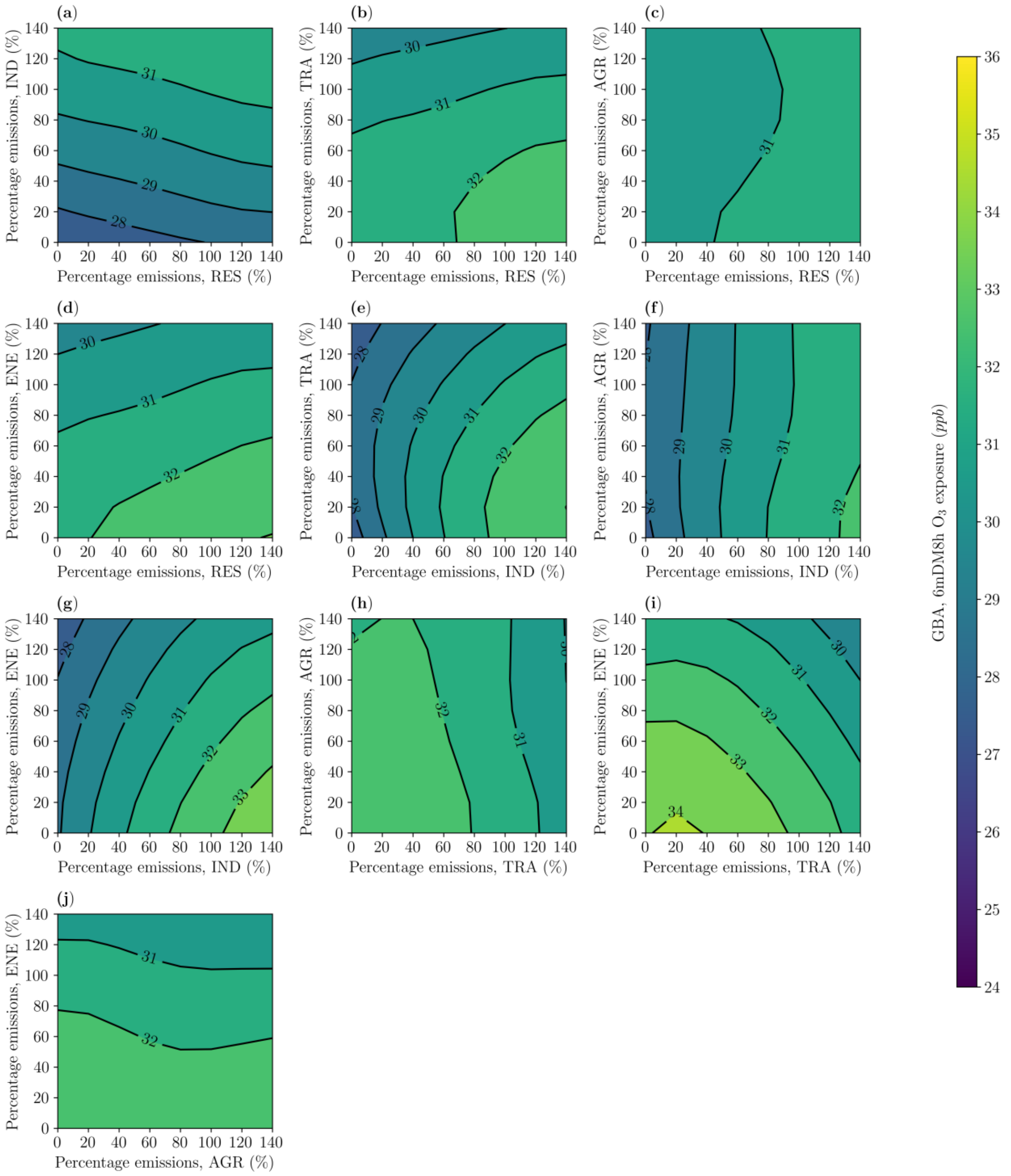

Figure S23: The impact of variations in two emission sectors on ozone (O_3, maximum 6−monthly−mean daily−maximum 8−hour, 6mDM8h) exposure for Guangdong−Hong Kong−Macau Greater Bay Area (GBA) from (a) residential (RES) and industry (IND), (b) RES and land transport (TRA), (c) RES and agriculture (AGR), (d) RES and power generation (ENE), (e) IND and TRA, (f) IND and AGR, (g) IND and ENE, (h) TRA and AGR, (i) TRA and ENE, and (j) AGR and ENE emissions.

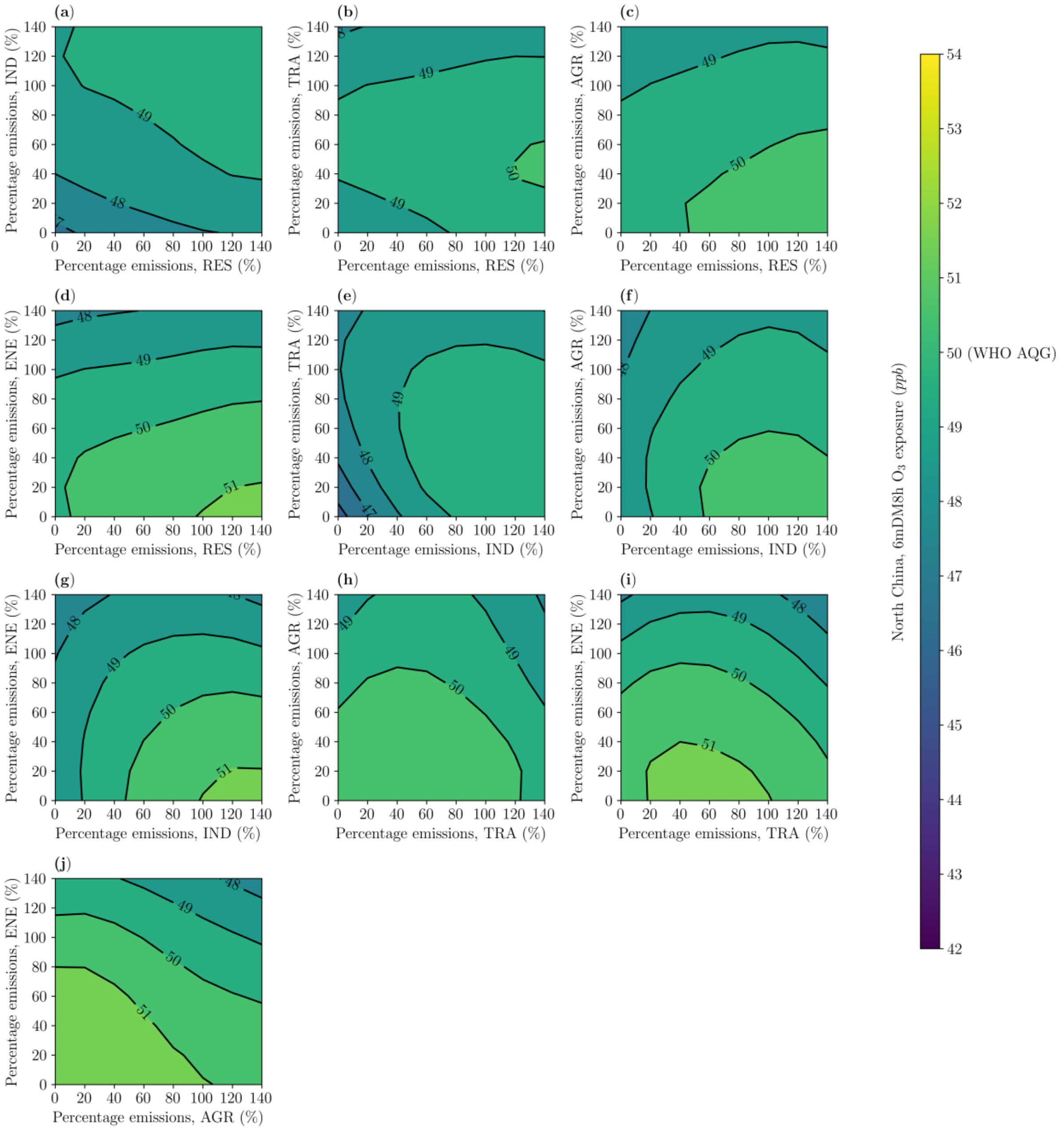

Figure S24: The impact of variations in two emission sectors on ozone (O_3, maximum 6−monthly−mean daily−maximum 8−hour, 6mDM8h) exposure for North China from (a) residential (RES) and industry (IND), (b) RES and land transport (TRA), (c) RES and agriculture (AGR), (d) RES and power generation (ENE), (e) IND and TRA, (f) IND and AGR, (g) IND and ENE, (h) TRA and AGR, (i) TRA and ENE, and (j) AGR and ENE emissions. Air quality targets shown for the World Health Organization's (WHO) Air Quality Guideline (AQG, 50 ppb).

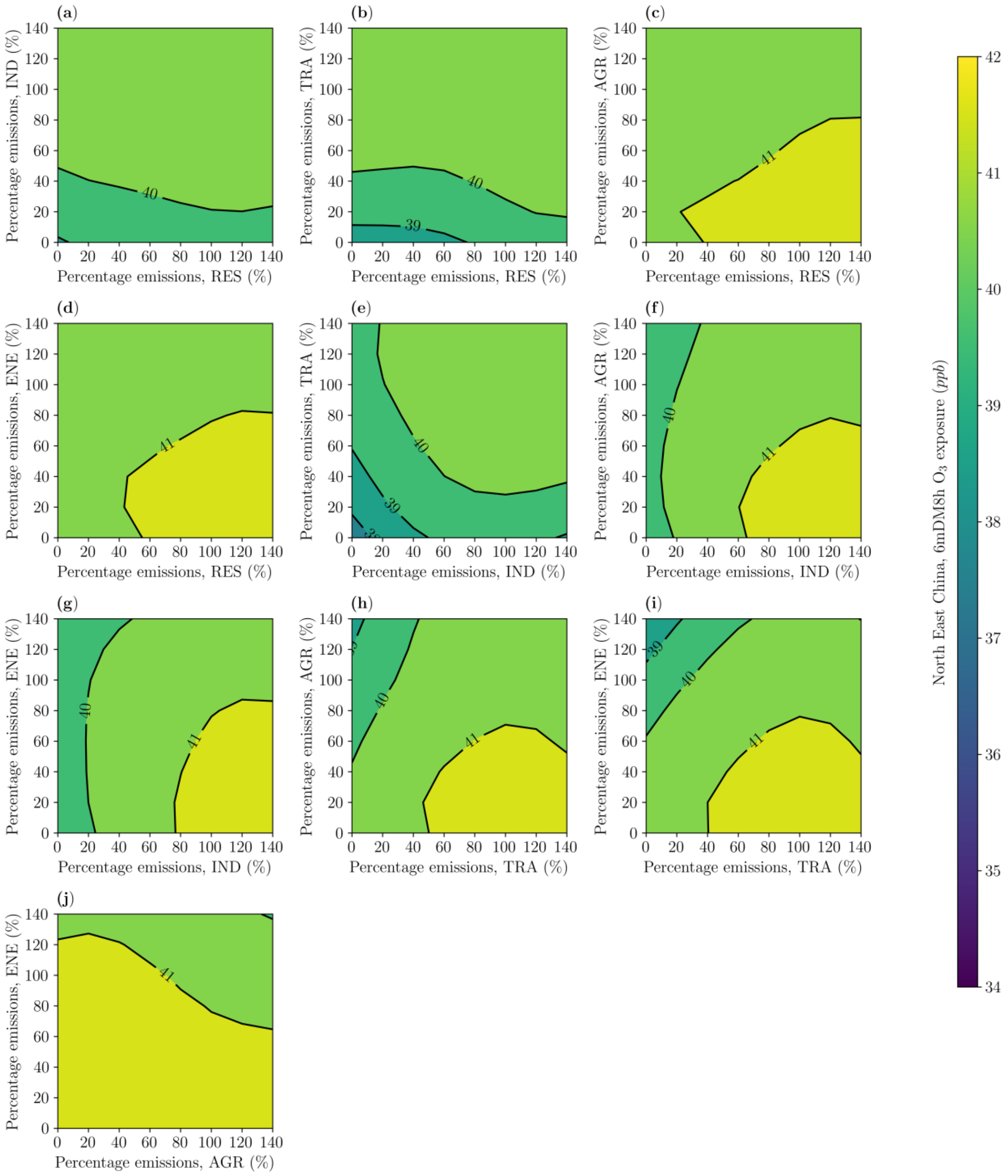

Figure S25: The impact of variations in two emission sectors on ozone (O_3, maximum 6−monthly−mean daily−maximum 8−hour, 6mDM8h) exposure for North East China from (a) residential (RES) and industry (IND), (b) RES and land transport (TRA), (c) RES and agriculture (AGR), (d) RES and power generation (ENE), (e) IND and TRA, (f) IND and AGR, (g) IND and ENE, (h) TRA and AGR, (i) TRA and ENE, and (j) AGR and ENE emissions.

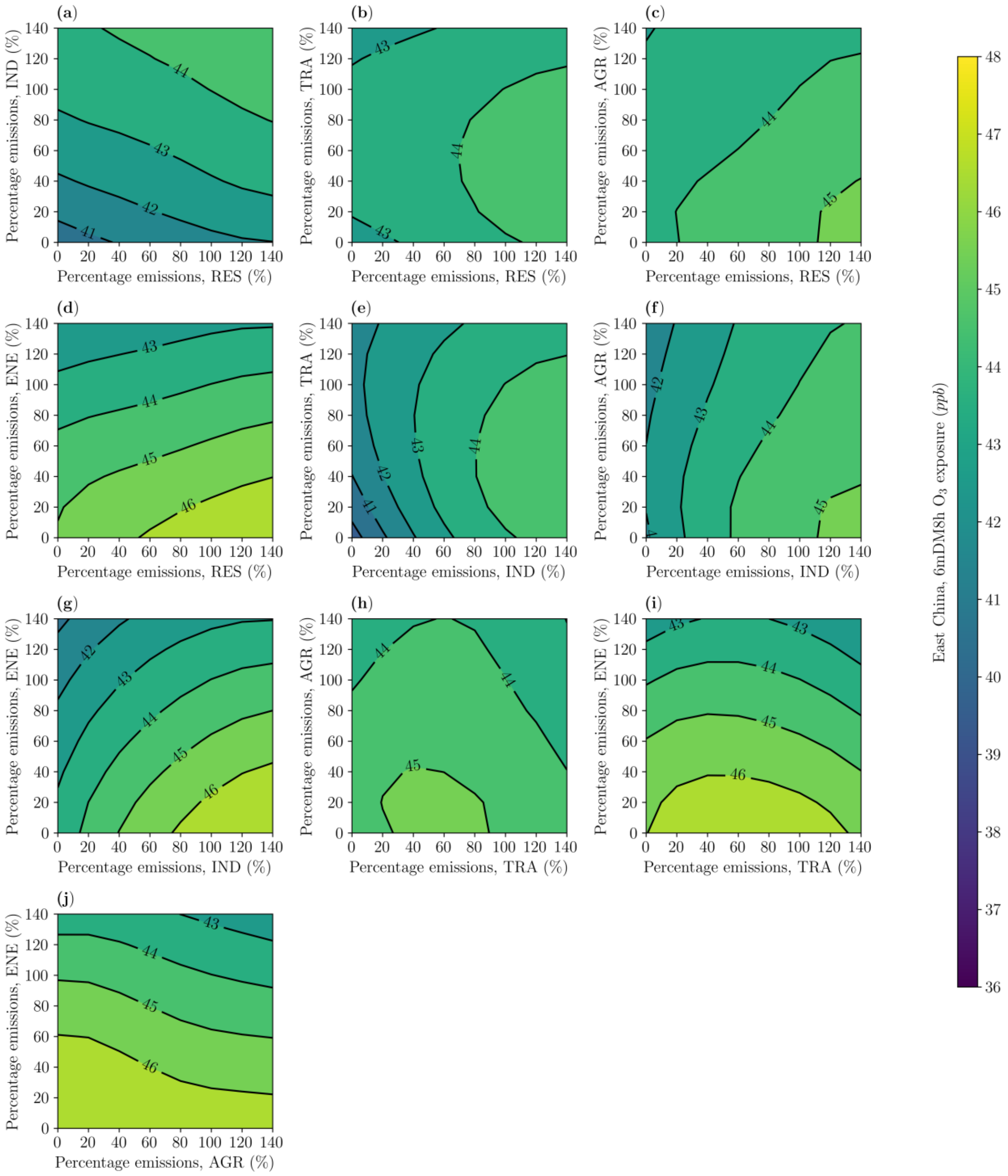

Figure S26: The impact of variations in two emission sectors on ozone (O_3, maximum 6−monthly−mean daily−maximum 8−hour, 6mDM8h) exposure for East China from (a) residential (RES) and industry (IND), (b) RES and land transport (TRA), (c) RES and agriculture (AGR), (d) RES and power generation (ENE), (e) IND and TRA, (f) IND and AGR, (g) IND and ENE, (h) TRA and AGR, (i) TRA and ENE, and (j) AGR and ENE emissions.

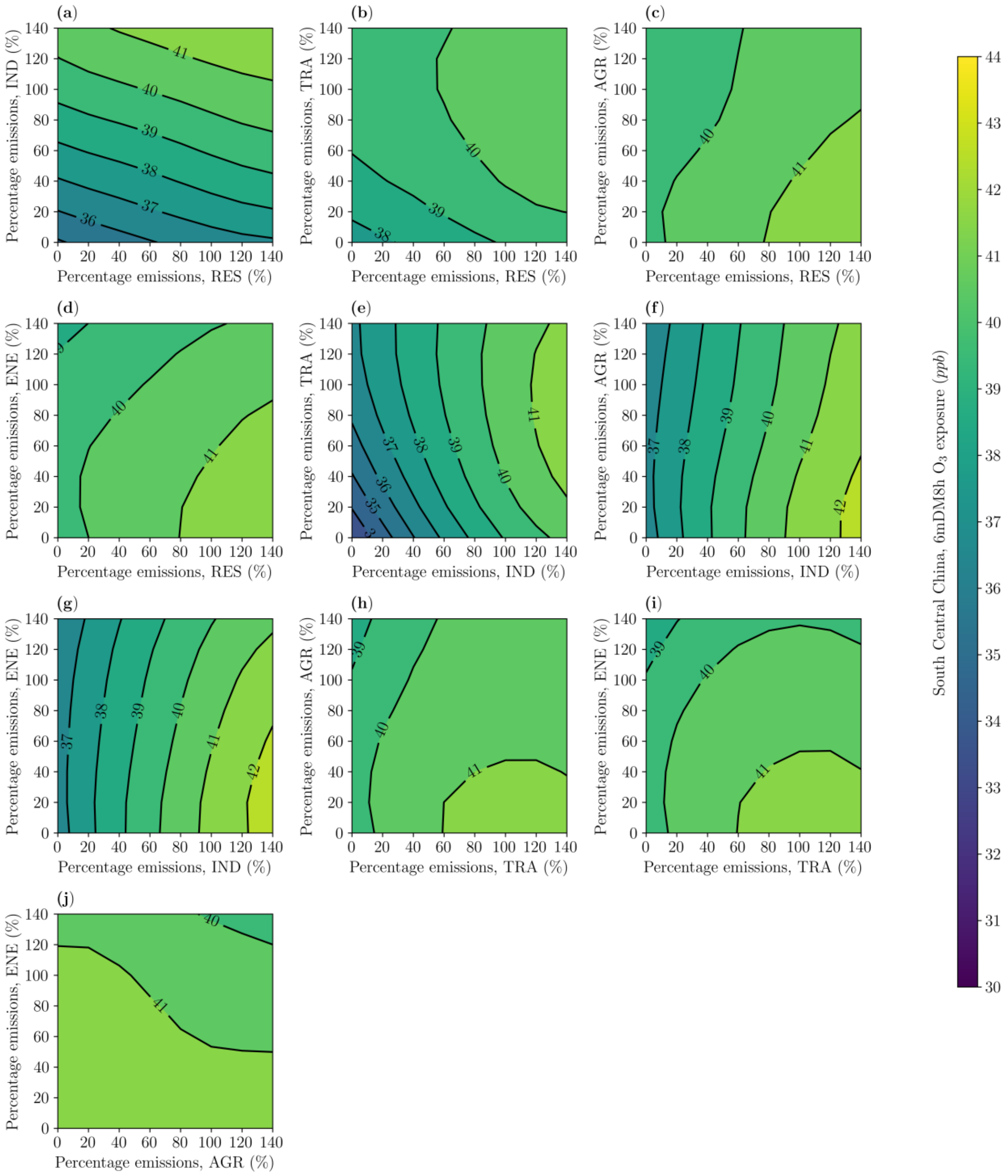

Figure S27: The impact of variations in two emission sectors on ozone (O_3, maximum 6−monthly−mean daily−maximum 8−hour, 6mDM8h) exposure for South Central China from (a) residential (RES) and industry (IND), (b) RES and land transport (TRA), (c) RES and agriculture (AGR), (d) RES and power generation (ENE), (e) IND and TRA, (f) IND and AGR, (g) IND and ENE, (h) TRA and AGR, (i) TRA and ENE, and (j) AGR and ENE emissions.

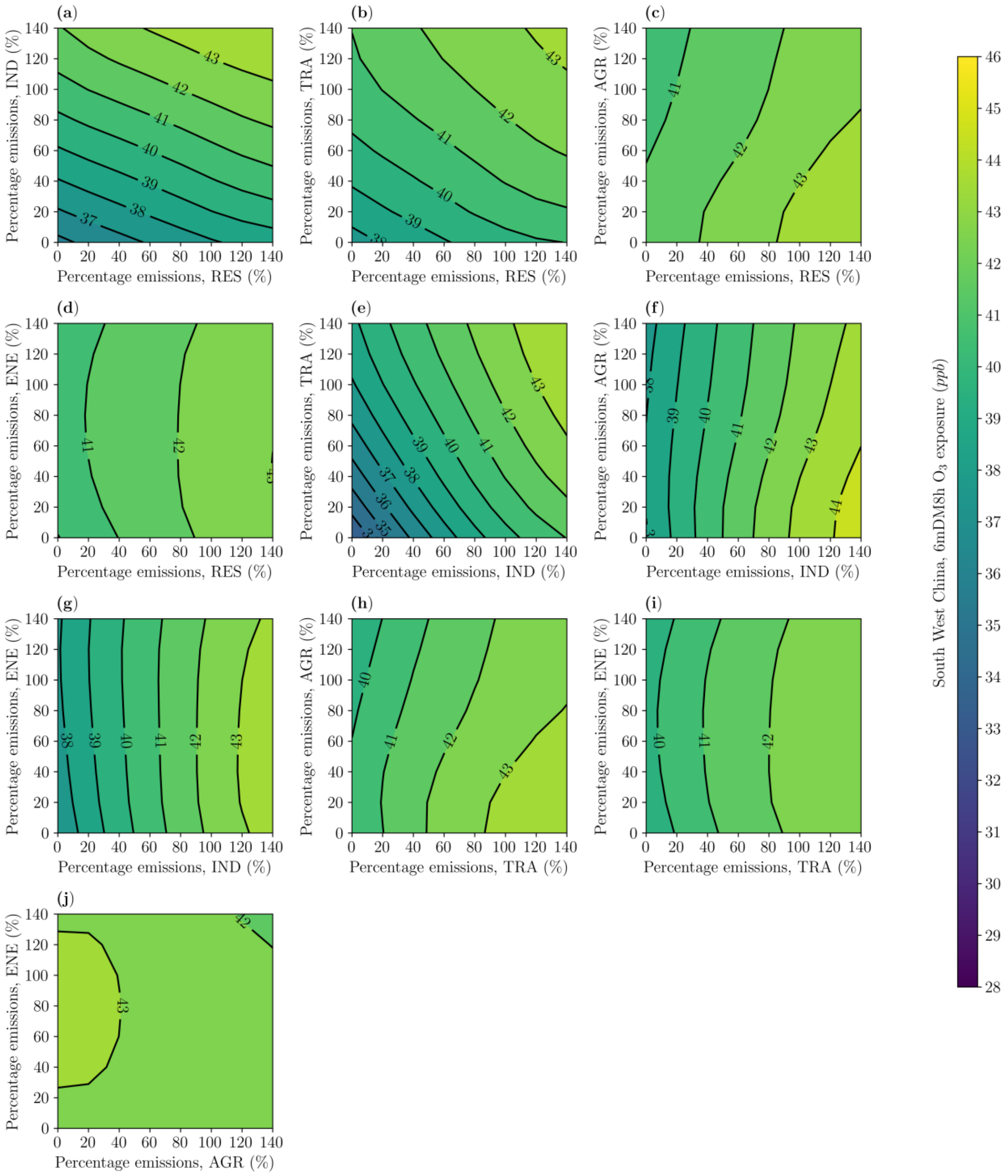

Figure S28: The impact of variations in two emission sectors on ozone (O_3, maximum 6−monthly−mean daily−maximum 8−hour, 6mDM8h) exposure for South West China from (a) residential (RES) and industry (IND), (b) RES and land transport (TRA), (c) RES and agriculture (AGR), (d) RES and power generation (ENE), (e) IND and TRA, (f) IND and AGR, (g) IND and ENE, (h) TRA and AGR, (i) TRA and ENE, and (j) AGR and ENE emissions.

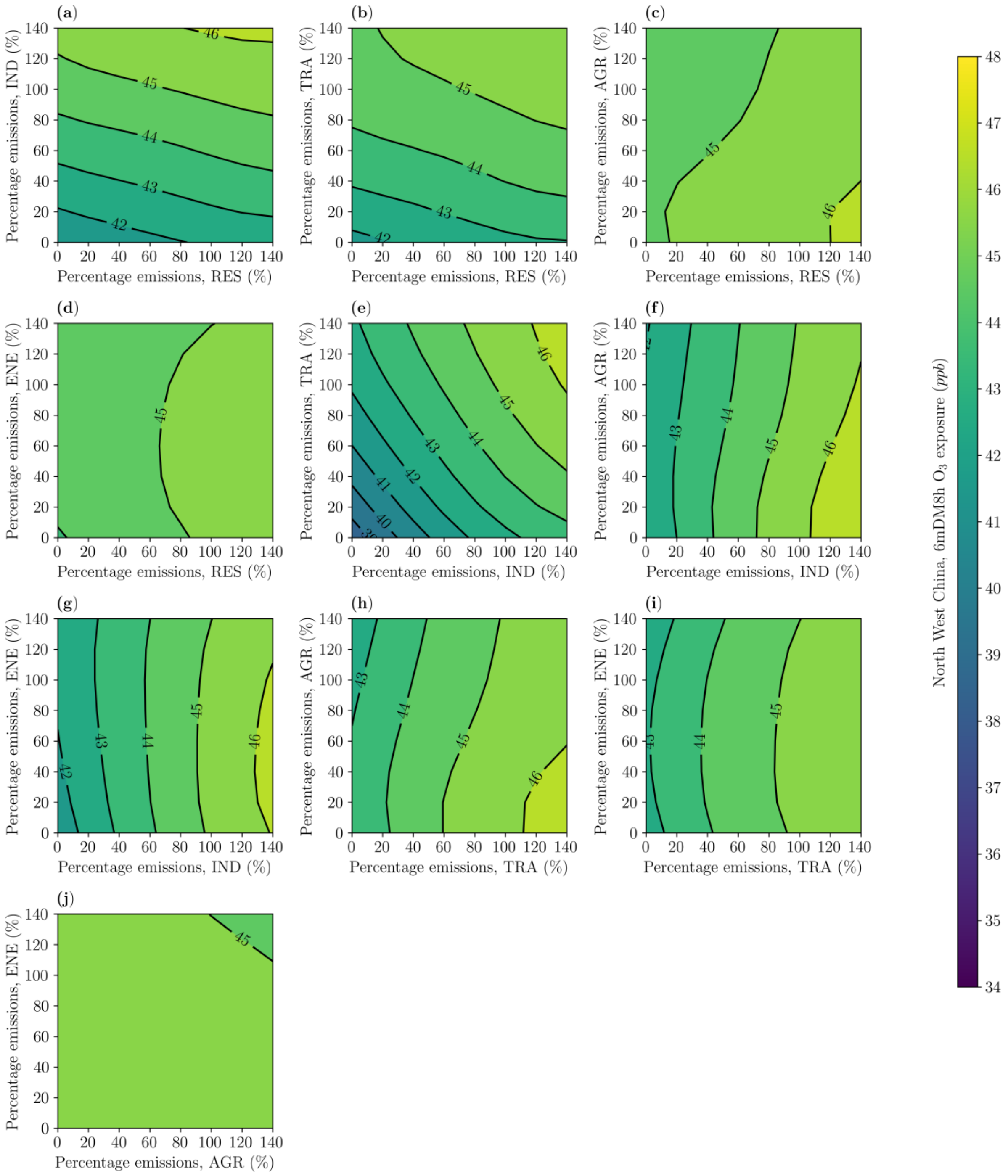

Figure S29: The impact of variations in two emission sectors on ozone (O_3, maximum 6−monthly−mean daily−maximum 8−hour, 6mDM8h) exposure for North West China from (a) residential (RES) and industry (IND), (b) RES and land transport (TRA), (c) RES and agriculture (AGR), (d) RES and power generation (ENE), (e) IND and TRA, (f) IND and AGR, (g) IND and ENE, (h) TRA and AGR, (i) TRA and ENE, and (j) AGR and ENE emissions.

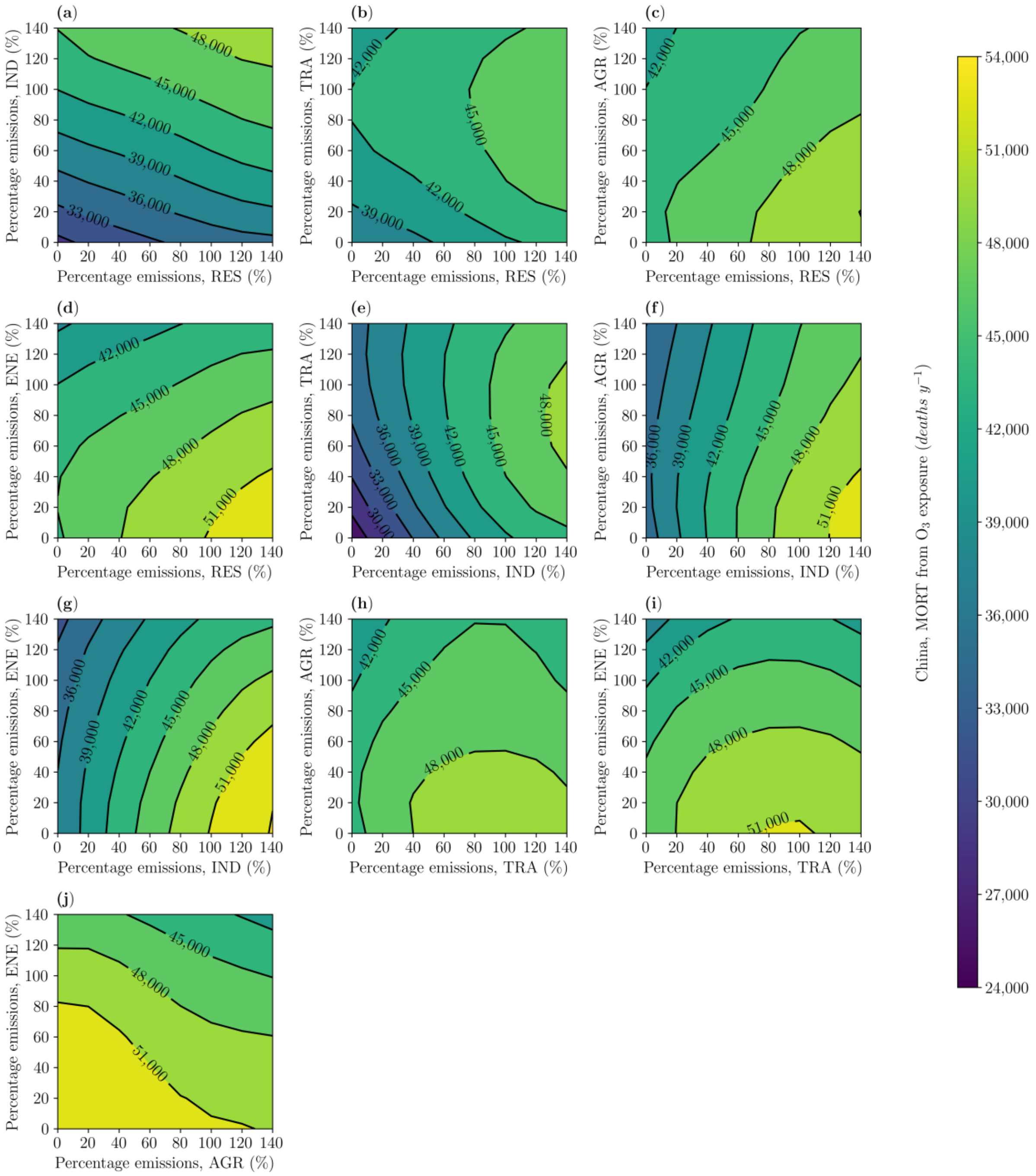

Figure S30: The impact of variations in two emission sectors on the disease burden (premature mortalities, MORT, per year) from ozone (O_3, maximum 6−monthly−mean daily−maximum 8−hour, 6mDM8h) exposure for China from (a) residential (RES) and industry (IND), (b) RES and land transport (TRA), (c) RES and agriculture (AGR), (d) RES and power generation (ENE), (e) IND and TRA, (f) IND and AGR, (g) IND and ENE, (h) TRA and AGR, (i) TRA and ENE, and (j) AGR and ENE emissions.

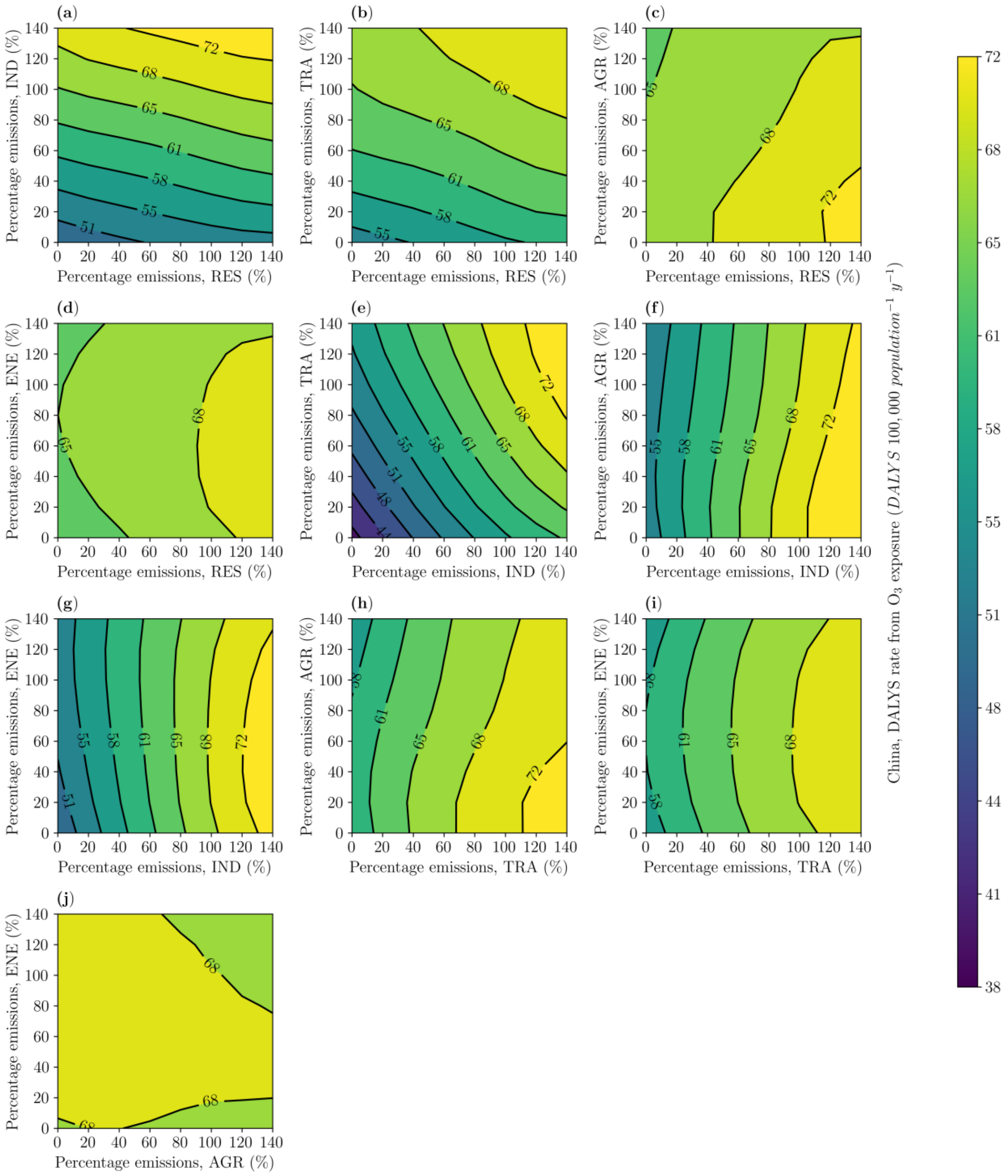

Figure S31: The impact of variations in two emission sectors on the disease burden (rate of disability−adjusted life years, DALYs, per 100,000 people per year) from ozone (O_3, maximum 6−monthly−mean daily−maximum 8−hour, 6mDM8h) exposure for China from (a) residential (RES) and industry (IND), (b) RES and land transport (TRA), (c) RES and agriculture (AGR), (d) RES and power generation (ENE), (e) IND and TRA, (f) IND and AGR, (g) IND and ENE, (h) TRA and AGR, (i) TRA and ENE, and (j) AGR and ENE emissions.

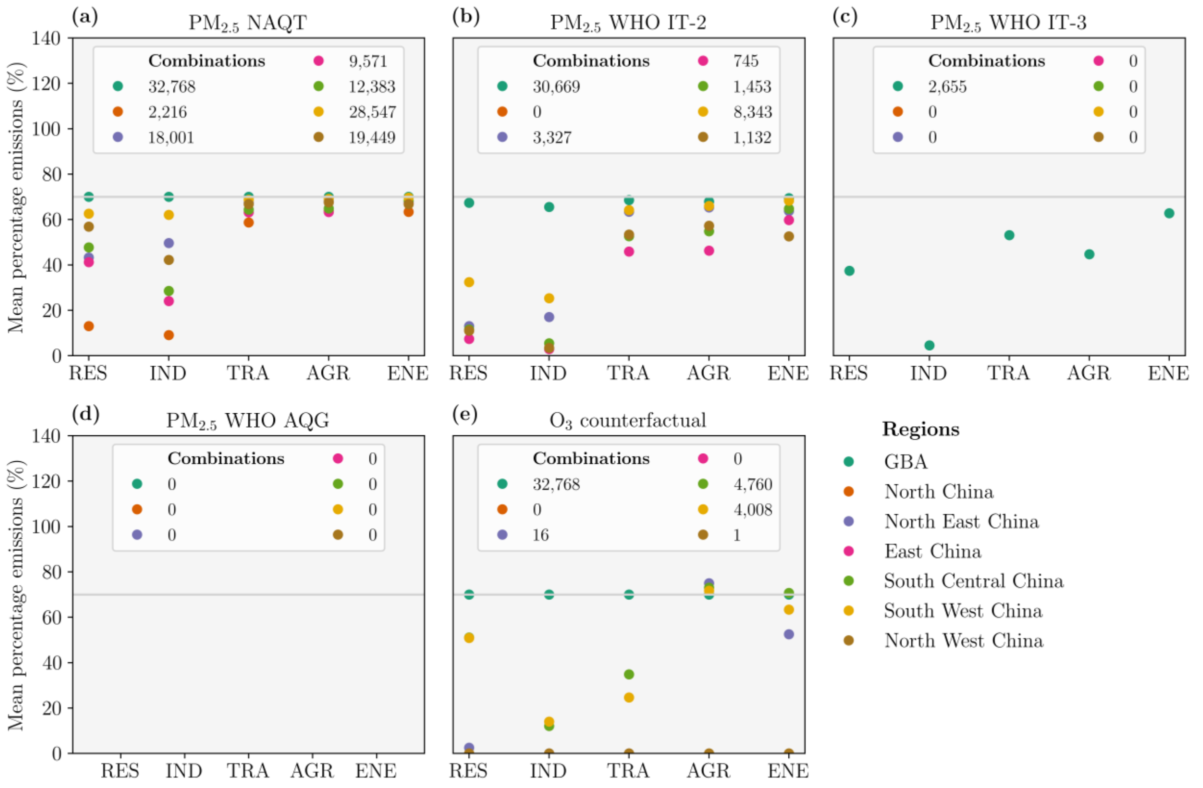

Figure S32: Mean emission configurations that meet air quality targets regionally across China. The number of emission combinations that meet the air quality targets are given per target and region. The horizontal line at 70% emissions is the average emissions if all combinations meet the air quality target. Targets are the (a) National Air Quality Target (NAQT, 35 µg m^{-3}) for ambient fine particulate matter ($PM_{2.5}$) concentrations, (b) World Health Organization (WHO) Interim Target 2 (IT−2, 25 µg m^{-3}) for $PM_{2.5}$ concentrations, (c) WHO Interim Target 3 (IT−3, 15 µg m^{-3}) for $PM_{2.5}$ concentrations, (d) WHO Air Quality Guideline (AQG, 5 µg m^{-3}) for $PM_{2.5}$ concentrations, and (e) counterfactual exposure level of no excess risk for ozone (O_3, 35.7 ppb) concentrations. Regions are Guangdong−Hong Kong−Macau Greater Bay Area (GBA) and North, North East, East, South Central, South West, and North West China. Sectors are residential (RES), industrial (IND), land transport (TRA), agricultural (AGR), and power generation (ENE) emissions.

References

Conibear, L., Reddington, C. L., Silver, B. J., Chen, Y., Arnold, S. R., & Spracklen, D. V. (2022). Supplementary Data: Sensitivity of Air Pollution Exposure and Disease Burden to Emission Changes in China using Machine Learning Emulation. University of Leeds. [Dataset]. https://doi.org/doi.org/10.5518/1055
